# MYB96 recruits the HDA15 protein to suppress negative regulators of ABA signaling in *Arabidopsis*

**DOI:** 10.1038/s41467-019-09417-1

**Published:** 2019-04-12

**Authors:** Hong Gil Lee, Pil Joon Seo

**Affiliations:** 1 Department of Chemistry, Seoul National University, Seoul, 08826 Republic of Korea; 2 Plant Genomics and Breeding Institute, Seoul National University, Seoul, 08826 Republic of Korea

## Abstract

Unlike activation of target genes in response to abscisic acid (ABA), how MYB96 transcription factor represses ABA-repressible genes to further enhance ABA responses remains unknown. Here, we show MYB96 interacts with the histone modifier HDA15 to suppress negative regulators of early ABA signaling. The MYB96-HDA15 complex co-binds to the promoters of a subset of *RHO GTPASE OF PLANTS* (*ROP*) genes, *ROP6*, *ROP10*, and *ROP11*, and represses their expression by removing acetyl groups of histone H3 and H4 from the cognate regions, particularly in the presence of ABA. In support, *HDA15*-deficient mutants display reduced ABA sensitivity and are susceptible to drought stress with derepression of the *ROP* genes, as observed in the *myb96-1* mutant. Biochemical and genetic analyses show that MYB96 and HDA15 are interdependent in the regulation of *ROP* suppression. Thus, MYB96 confers maximal ABA sensitivity by regulating both positive and negative regulators of ABA signaling through distinctive molecular mechanisms.

## Abstract (web summary)

MYB96 can regulate both positive and negative regulators of ABA signaling to maximize plant drought tolerance. Here, the authors show that MYB96 represses expression of ABA negative regulators in *Arabidopsis* by interacting with HDA15 and promoting histone deacetylation at the cognate regions.

## Introduction

Sessile plants have evolved a remarkable ability to adapt to the environment, which is largely mediated by abscisic acid (ABA)-dependent signaling pathways^1,2^. ABA is recognized by plasma membrane-localized cell receptors, including PYRABACTIN RESISTANCE/PYRABACTIN RESISTANCE-LIKE/REGULATORY COMPONENT OF ABSCISIC ACID RECEPTOR (PYR/PYL/RCAR) family proteins^3,4^. These receptors constitute early ABA signaling modules, together with PROTEIN PHOSPHATASE 2Cs (PP2Cs) and subfamily 2 SNF1-RELATED PROTEIN KINASEs (SnRK2s)^5,6^. In the presence of ABA, the PYR receptors inhibit PP2C activity and thereby derepress the catalytic functions of SnRK2^5,7^. A number of transcription factors from bZIP, NAC, MYC, and MYB families are then activated to stimulate downstream ABA responses^8,9^.

The R2R3-type MYB96 transcription factor is a master transcriptional regulator that mediates a variety of plant responses to ABA, including seed germination, drought tolerance, stomatal conductance, lateral root development, hormone biosynthesis, anthocyanin accumulation, and cuticular wax biosynthesis^10–15^. The ABA-inducible MYB96 transcription factor binds to the promoters of many ABA-responsive genes and usually activates expression to optimize plant growth and fitness under unfavorable conditions^10–12^. Notably, like many plant transcription factors^16^, MYB96 has also been suspected to bind to the promoters of ABA-repressed genes, such as negative regulators of ABA signaling, and sometimes functions as a transcriptional repressor to further enhance plant responses to environmental stresses^17–19^. However, it remains unclear how MYB96 represses gene expression and what trans-factor mediates MYB96-dependent gene repression.

Two classes of signaling G proteins exist in eukaryotes: heterotrimeric G proteins and monomeric Ras/Ras-like small GTPases^20,21^. In particular, Rho family GTPases, which belong to the Ras superfamily, are likely major molecular switches that mediate diverse cellular responses to multiple extracellular signals in plants^22^. ROP (Rho of Plants)/RAC is the only subfamily of Rho family GTPases in plants^20^, and the *Arabidopsis* genome contains 11 ROP GTPases with four phylogenetic groups: group I (ROP8), group II (ROP9–ROP11), group III (ROP7), and group IV (ROP1–ROP6)^23,24^. A subset of ROP GTPases plays negative roles in ABA responses in *Arabidopsis*. For instance, overexpression of *ROP2* and *ROP6* reduces ABA sensitivity during seed germination and stomatal closure^25,26^. The *rop10* mutation enhances ABA-induced suppression of seed germination, primary root growth, and lateral root formation with increased expression of ABA-responsive genes^27^. ROP11 is also a negative regulator of multiple ABA responses, including seed germination, early seedling growth, stomatal closure, and drought responses^28^. Further, ROP11 acts at an early step of ABA signaling by interacting with ABI1, a receptor component, at the plasma membrane and protecting ABI1 phosphatase activity from inhibition by the ABA receptor complex^29^. The ROP signaling networks coordinate many downstream pathways to ensure large repertoires of output responses.

In this study, we find that MYB96 negatively regulates a subset of *ROP* genes, which repress ABA signaling at the early stages of signal transduction. Repression of *ROP* genes by MYB96 requires the histone modifier HDA15. The MYB96 transcription factor specifically binds to promoters of the *ROP6*, *ROP10*, and *ROP11* genes and recruits the HDA15 protein to catalyze H3 and H4 deacetylation at cognate sites. While MYB96 alone acts as a transcriptional activator, the MYB96–HDA15 complex can perform repressive functions in gene regulation. These results indicate that MYB96 acts as both a transcriptional activator and repressor to enhance ABA signaling.

## Results

### MYB96 interacts with HDA15

MYB96 is a pivotal regulator of ABA signaling and mediates a variety of physiological responses required for environmental stress adaptation in plants^10–14,30,31^. Consistent with MYB96 being a transcriptional activator, a number of ABA-inducible genes are primary targets of MYB96^11,12,14,30,31^. However, accumulating evidence suggests that many negative regulators of ABA signaling may also be repressed in the *myb96-ox* overexpression mutant^12^, suggesting that MYB96 may further enhance ABA responses by directly repressing ABA-repressed genes.

**Fig. 1:**
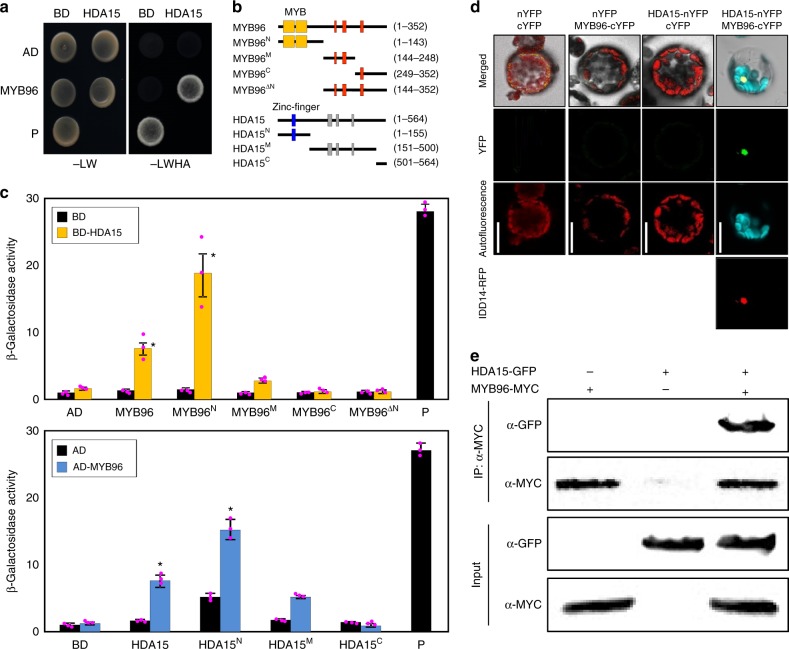
Interaction of MYB96 with HDA15. **a** Yeast-two-hybrid (Y2H) assays. Y2H assays were performed with the HDA15 protein fused to the DNA binding domain (BD) of GAL4 and MYB96 fused to the transcriptional activation domain (AD) of GAL4. Interaction of the two proteins was examined by cell growth on selective media. -LWHA indicates Leu, Trp, His, and Ade drop-out plates. -LW indicates Leu and Trp drop-out plates. GAL4 was used as a positive control (P). **b** Deletion constructs of HDA15 and MYB96. Numbers indicate residue positions. Red and gray rectangles represent low complexity regions and histone deacetylase domains, respectively. **c** Interaction domain mapping. β-Galactosidase (β-Gal) activity was quantified after growing yeast strains in liquid culture with *o*-nitrophenyl-β-d-galactopyranoside as a substrate. Three independent measurements of β-galactosidase activities were averaged and statistically analyzed by a Student’s *t* test (*P < 0.05). Bars indicate the standard error of the mean. **d** Bimolecular fluorescence complementation assays. Partial yellow fluorescent protein fusion constructs containing either HDA15 or MYB96 were transiently co-expressed in *Arabidopsis* protoplasts. The IDD14-RFP construct was used as a nuclear localization marker. Scale bars = 20 μm. **e** Coimmunoprecipitation (Co-IP) assays. *Agrobacterium tumefaciens* cells containing MYB96-MYC and HDA15-GFP constructs were injected into 3-week-old *Nicotiana benthamiana* leaves. Epitope-tagged proteins were detected immunologically using corresponding antibodies. IP immunoprecipitation. Source data are provided as a Source Data file

We postulated that MYB96 may recruit additional trans-factor(s) to repress gene expression. To obtain clues to the identities of potential interacting factors, we performed yeast-two-hybrid (Y2H) screening. Preliminary analysis identified the HDA15 protein as an interaction partner of MYB96. To validate this observation, the MYB96 protein fused with the GAL4 activation domain (AD) was coexpressed in yeast cells with the full-length HDA15 protein fused with the GAL4 DNA binding domain (BD). Growth on selective medium showed that MYB96 interacts with HDA15 (Fig. 1a). Deletion constructs further revealed that an N-terminal fragment of MYB96 containing the R2R3-MYB DNA BD was responsible for interaction with the N-terminal fragment of HDA15 (Fig. 1b, c). A zinc-finger motif in HDA15 might contribute to mediating the protein–protein interactions. Interactions of MYB96 and HDA15 were mutually specific. No other HDAC protein associated with MYB96 (Supplementary Figure 1). Likewise, other MYB transcription factors involved in ABA responses did not physically associate with HDA15 (Supplementary Figure 2).

To further support the in vivo interactions between MYB96 and HDA15, we conducted bimolecular fluorescence complementation (BiFC) analysis. The *HDA15* sequence was translationally fused in-frame to the 5’-end of a gene sequence encoding the N-terminal half of yellow fluorescent protein (nYFP), and the *MYB96* gene was fused in-frame to the 5’-end of a sequence encoding the C-terminal half of YFP (cYFP). The fusion constructs were then transiently coexpressed in *Arabidopsis* protoplasts. As a result, strong yellow fluorescence was detected in the nucleus for the MYB96–HDA15 combination, while no fluorescence signal was observed when empty vectors were coexpressed (Fig. 1d and Supplementary Figure 3).

We confirmed in planta interactions of MYB96 and HDA15 by coimmunoprecipitation (Co-IP). 35S:*MYB96-MYC* and 35S:*HDA15-GFP* constructs were coexpressed in leaf cells of *Nicotiana benthamiana* by *Agrobacterium* infiltration. Total protein extracts were immunoprecipitated with an anti-MYC antibody, which mainly pulls down the MYB96-MYC protein. Then immunoblot analysis was performed with an anti-green fluorescent protein (anti-GFP) antibody and the HDA15 fusion protein was detected (Fig. 1e), indicating in planta interactions between MYB96 and HDA15.

### HDA15 promotes ABA signaling

**Fig. 2:**
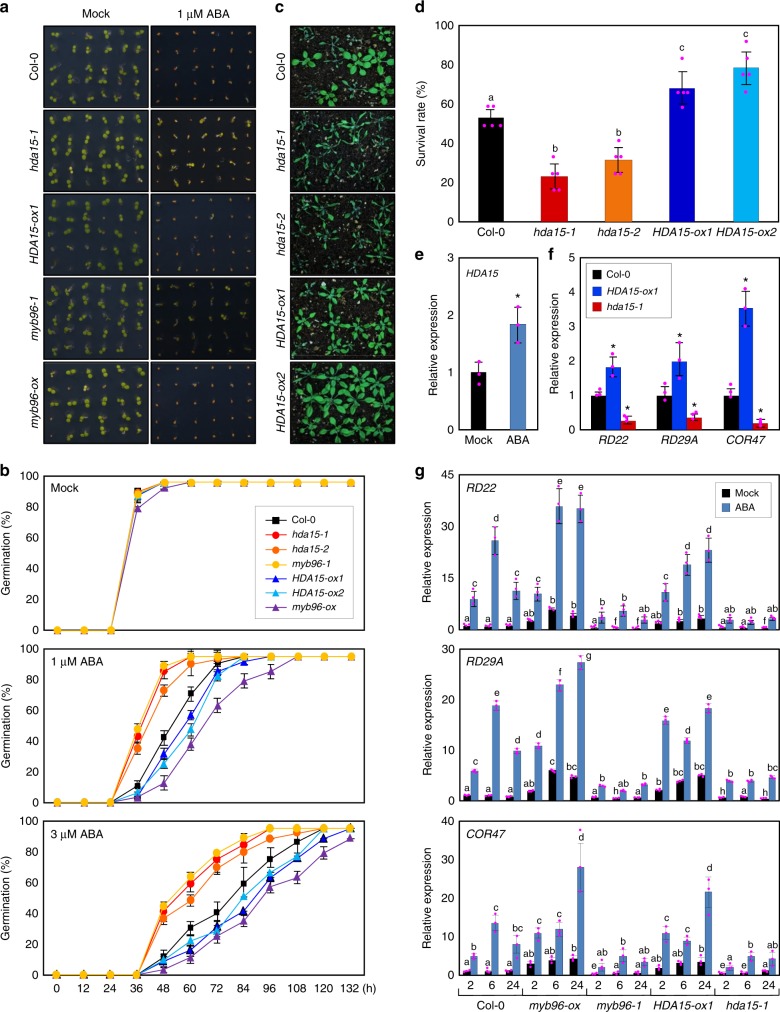
Abscisic acid (ABA) sensitivity of *HDA15-ox* transgenic and *hda15*-deficient plants. **a** Altered ABA sensitivity of *HDA15-ox* and *hda15-1* seeds. Seeds were germinated and grown on MS-medium supplemented with 1 μM ABA under long-day (LD) conditions. Photographs were taken 4 days after cold stratification (DAC). *myb96-1* and *myb96-ox* mutant seeds were used as controls. **b** Germination percentage. The percentage of seed germination of the indicated genotypes grown on different concentrations of ABA was quantified after the end of stratification (h). Radicle emergence was used as a morphological marker for germination. At least 30–50 seeds per genotype were measured in each replicate. Biological triplicates were averaged. Bars indicate the standard deviation of the mean. **c** Drought tolerance of *HDA15-ox* and *hda15* mutant plants. **d** Survival rate. Three independent biological replicates were averaged, and statistical significance of the measurements was analyzed by analysis of variance (ANOVA) (one-way ANOVA with Fisher’s post hoc test, *P < 0.05). Different letters represent a significant difference at *P* < 0.05. Bars indicate the standard deviation of the mean. **e** Effects of ABA on *HDA15* expression. Two-week-old plants grown under LDs were transferred to half-strength MS-liquid medium supplemented with 20 μM ABA and incubated for 6 h. Transcript accumulation was analyzed by quantitative real-time reverse transcriptase-PCR. Three independent biological replicates were averaged, and statistical significance of the measurements was analyzed by a Student’s *t* test (*P < 0.05). Bars indicate the standard error of the mean. **f** Expression of ABA-responsive genes in *HDA15-ox* and *hda15-1*. Three independent biological replicates were averaged, and statistically significant differences between the wild-type and transgenic or mutant plants are indicated by asterisks (Student’s *t* test, *P *<* 0.05). **g** HDA15 regulation of ABA induction of ABA marker genes. Two-week-old seedlings grown under LDs were transferred to MS-liquid medium supplemented with 20 μM ABA and incubated for the indicated time period (h). Three independent biological replicates were averaged, and statistical significance of the measurements was analyzed by ANOVA (one-way ANOVA with Fisher’s post hoc test, *P < 0.05). Different letters represent a significant difference at *P* < 0.05. Source data are provided as a Source Data file

Our results raised the possibility that HDA15 is involved in ABA signaling, possibly in connection with MYB96-regulated signaling networks. Thus we obtained T-DNA insertional mutants, *hda15-1* and *hda15-2*, and produced *HDA15*-overexpressing *HDA15-ox* transgenic plants (Supplementary Figure 4) to examine ABA sensitivity in germination rate. In the absence of ABA, the germination rate of *hda15*-deficient mutant and *HDA15-ox* transgenic seeds was indistinguishable from wild-type seeds (Fig. 2a, b). However, in the presence of ABA, *hda15* mutant seeds exhibited reduced sensitivity to ABA (Fig. 2a, b), similar to *myb96-1* mutant seeds^10,14^. In contrast, higher ABA sensitivity was observed in *HDA15-ox* and *myb96-ox* transgenic seeds during germination (Fig. 2a, b). The differential sensitivity was more pronounced at higher concentrations of ABA (Supplementary Figure 5). Moreover, plant tolerance to drought stress, which is mainly conferred by ABA^32^, was also influenced by HDA15 (Fig. 2c). The *hda15* mutant plants exhibited higher susceptibility to water deficit, whereas *HDA15-ox* transgenic plants were more tolerant to drought stress than wild-type plants (Fig. 2c, d), similar to *MYB96*-misexpressing plants (Supplementary Figure 6), suggesting that HDA15 positively regulates ABA responses.

Consistent with the positive role of HDA15 in ABA responses, the *HDA15* gene was induced by ABA treatment (Fig. 2e and Supplementary Figure 7). Abiotic stress factors, including osmotic and cold stress, also promoted *HDA15* expression (Supplementary Figure 7). In addition, the expression of some ABA-responsive genes was influenced by HDA15 activity. Even in the absence of exogenous ABA application, *HDA15-ox* transgenic plants exhibited increased transcript accumulation of ABA-responsive marker genes such as *RESPONSIVE TO DESSICATION 22* (*RD22*), *RD29A*, and *COLD-REGULATED 47* (*COR47*)^33^, whereas the *hda15-1* mutant showed reduced transcript accumulation of these genes (Fig. 2f). Furthermore, ABA induction of the *RD* and *COR* genes was also impaired in the *hda15-1* and *myb96-1* mutants, regardless of plant ages (Fig. 2g and Supplementary Figure 8). These results indicate that HDA15 is an ABA signaling component that promotes ABA responses.

### MYB96 and HDA15 act synergistically in ABA signaling

**Fig. 3:**
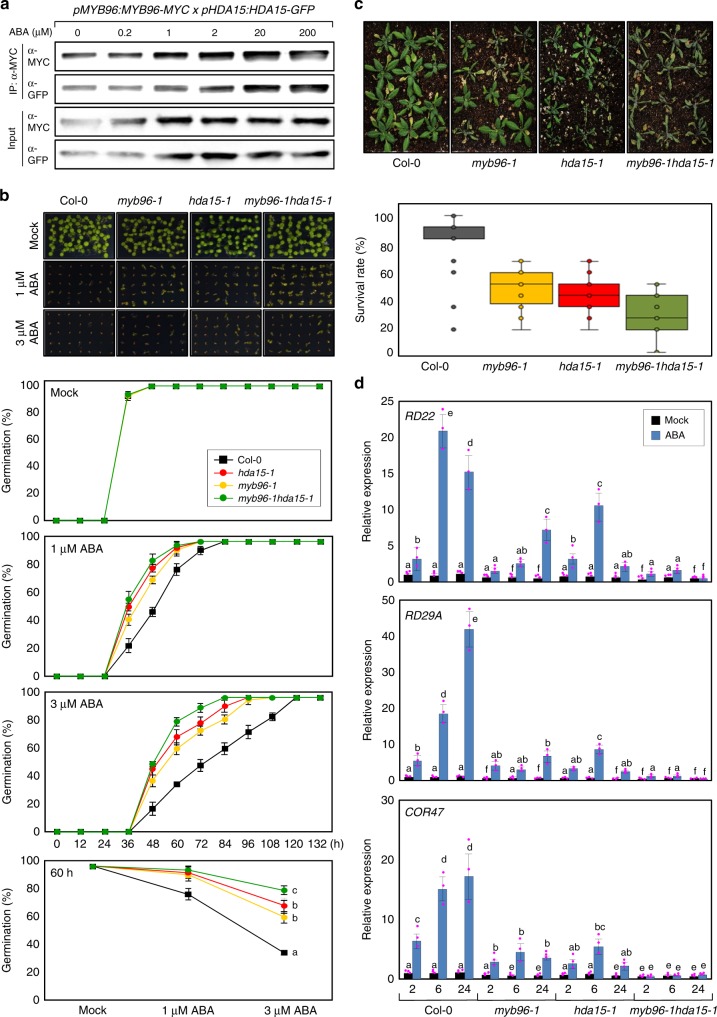
Synergistic interactions between MYB96 and HDA15. **a** Effects of abscisic acid (ABA) on interactions between MYB96 and HDA15. Two-week-old seedlings were used for treatment with different concentrations of ABA. Epitope-tagged proteins were precipitated and detected immunologically using the corresponding antibodies. IP immunoprecipitation. **b** Effects of ABA on seed germination in *myb96-1hda15-1*. The percentage of seed germination of the indicated genotypes grown on different concentrations of ABA was quantified after the end of stratification (h). Biological triplicates were averaged. Bars indicate the standard deviation of the mean. **c** Drought tolerance of *myb96-1hda15-1* plants. Two-week-old plants were treated for drought stress by stopping watering for additional 2 weeks. Three independent biological replicates were averaged. **d** ABA induction of ABA marker genes in *myb96-1hda15-1*. Two-week-old seedlings grown under long days were transferred to MS-liquid medium supplemented with 20 μM ABA and incubated for the indicated time period (h). Three independent biological replicates were averaged, and statistical significance of the measurements was analyzed by analysis of variance (ANOVA) (one-way ANOVA with Fisher’s post hoc test, *P < 0.05). Different letters represent a significant difference at *P* < 0.05. Source data are provided as a Source Data file

Considering that MYB96 interacts with HDA15 and that they have overlapping functions in ABA signaling, MYB96 and HDA15 may act together in the control of ABA sensitivity. We first examined whether the physical interaction between MYB96 and HDA15 is affected by ABA. *pMYB96:MYB96-MYC x pHDA15:HDA15-GFP* transgenic *Arabidopsis* plants were generated and used for Co-IP assays. Co-IP followed by immunoblot analysis revealed that MYB96 and HDA15 form a complex, and interaction was further enhanced as ABA concentration increases (Fig. 3a), suggesting that they work together especially at high concentrations of ABA.

We then analyzed the genetic relationship between MYB96 and HDA15. To this end, the *myb96-1hda15-1* double mutant was generated to analyze ABA responses including seed germination and drought tolerance. In the presence of ABA, seed germination rate was significantly delayed in wild type, whereas *myb96-1* and *hda15-1* mutant seeds exhibited reduced sensitivity to ABA (Fig. 3b). Notably, the *myb96-1hda15-1* double mutant showed further reduction in ABA sensitivity compared to *myb96-1* and *hda15-1* seeds (Fig. 3b), particularly at a high concentration of ABA (Fig. 3b). Similarly, the *myb96-1hda15-1* double mutant also exhibited a higher susceptibility to drought stress than *myb96-1* and *hda15-1* single mutants (Fig. 3c). The reduced sensitivity to ABA in *myb96-1hda15-1* was supported by expression of the *RD* and *COR* genes. ABA induction of *RD* and *COR* genes was compromised in *myb96-1* and *hda15-1* mutants and more profoundly in the *myb96-1hda15-1* double mutant (Fig. 3d and Supplementary Figure 8). These results indicate that MYB96 and HDA15 act synergistically to confer ABA sensitivity at least in the processes of seed germination and drought tolerance.

### MYB96 recruits HDA15 to the *ROP* promoters

**Fig. 4:**
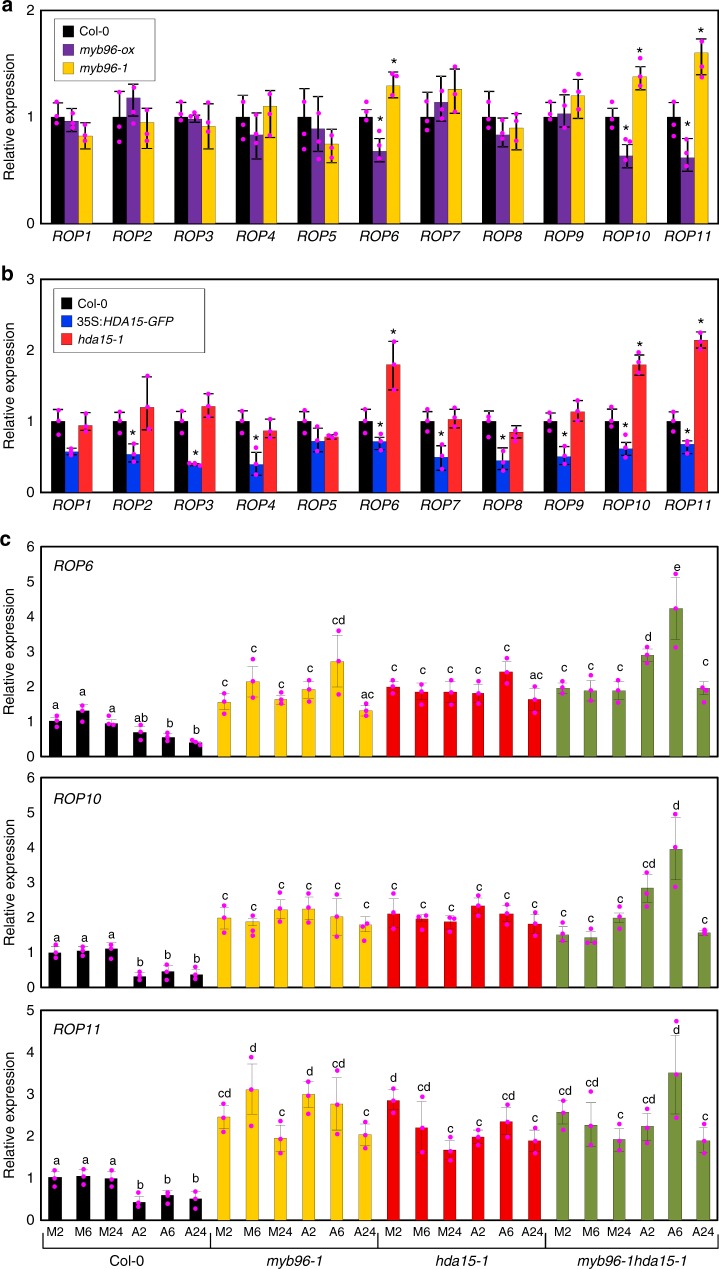
MYB96 and HDA15 regulate expression of Rho of Plants (*ROP*) genes. **a** Transcript accumulation of *ROP* genes in wild type, *myb96-ox*, and *myb96-1*. **b** Transcript accumulation of *ROP* genes in wild type, 35S:*HDA15-GFP*, and *hda15-1*. In **a**, **b**, 2-week-old seedlings grown under long days (LDs) were harvested for total RNA isolation. Statistically significant differences between the wild-type and transgenic or mutant plants (Student’s *t* test, *P *<* 0.05) are indicated by asterisks. **c** Effects of abscisic acid (ABA) on the expression of *ROP* genes in *myb96-1*, *hda15-1*, and *myb96-1hda15-1*. Two-week-old seedlings grown under LDs were transferred to MS-liquid medium supplemented with 20 μM ABA and incubated for the indicated time period (h). Transcript accumulation was analyzed by quantitative real-time reverse transcriptase-PCR. Three independent biological replicates were averaged, and statistical significance of the measurements was analyzed by analysis of variance (ANOVA) (one-way ANOVA with Fisher’s post hoc test, *P < 0.05). M mock, A ABA. Different letters represent a significant difference at *P* < 0.05. Source data are provided as a Source Data file

Previous microarray data assumed that MYB96 represses some negative regulators of ABA signaling^12^. To test this possibility, we examined transcript accumulation of negative regulators of ABA signaling in wild-type, *myb96-ox*, and *myb96-1* seedlings, including *ABI1*, *HIGHLY ABA-INDUCED PP2C GENE 2* (*HAI2*), *HYPERSENSITIVE TO ABA 1* (*HAB1*), *ABI3-INTERACTING PROTEIN 2* (*AIP2*), *PLANT U-BOX 9* (*PUB9*), *MORE AXILLARY GROWTH 2* (*MAX2*), *DWD HYPERSENSITIVE TO ABA 1* (*DWA1*), *DWA2*, and *ROP* genes. These genes are known as negative regulators of ABA signaling^34–37^, and their genetic mutants display enhanced ABA responses, such as delayed germination, reduced lateral root length, and increased drought tolerance^38–40^. Quantitative real-time reverse transcriptase-PCR (RT-qPCR) analysis showed that most of the genes were uninfluenced by MYB96, except for a subset of *ROP* genes, *ROP6*, *ROP10*, and *ROP11* (Fig. 4a; Supplementary Figure 9), which are known to negatively regulate early ABA signaling^26,29,41^. These *ROP* genes were specifically and significantly activated in *myb96-1* but reduced in *myb96-ox* (Fig. 4a).

Based on the mutual interaction of MYB96 and HDA15 and the transcriptional repressor activity of HDA15^42,43^, we hypothesized that MYB96 works together with HDA15 to repress the *ROP* genes. Although HDA15 broadly influenced the expression of *ROP* genes, the three *ROP* genes were specifically upregulated in the *hda15-1* mutant (Fig. 4b). To further estimate the relevance of MYB96 and HDA15 in ABA-dependent *ROP* suppression, we tested transcript levels of *ROP6*, *ROP10*, and *ROP11* in *myb96-1*, *hda15-1*, and *myb96-1hda15-1* mutant seedlings treated with ABA. The *ROP* genes were repressed in response to ABA in wild type, but ABA repression of *ROP* genes was impaired, and higher expression of *ROP* genes was observed in the *myb96-1*, *hda15-1*, and *myb96-1hda15-1* mutants (Fig. 4c). Although MYB96 and HDA15 were synergistic in the control of ABA responses (Fig. 3a–d), they both were required at least for the suppression of *ROP* expression in response to ABA (Fig. 4c).

**Fig. 5:**
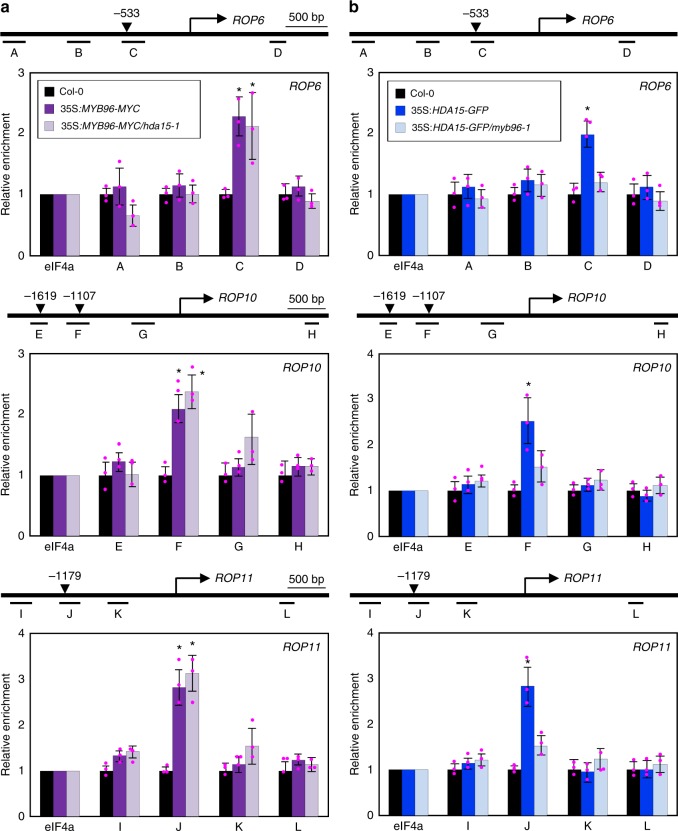
MYB96 and HDA15 co-bind to the *ROP* promoters. In **a**, **b**, putative MYB-binding sites are indicated by arrowheads. Black underbars indicate the regions of PCR amplification after chromatin immunoprecipitation (ChIP). ChIP-quantitative PCR assays were conducted to examine enrichment of MYB96 and HDA15 at *ROP* loci. Three independent biological replicates were averaged, and statistical significance of the measurements was analyzed by Student’s *t* test (*P < 0.05). Bars indicate the standard error of the mean. **a** Binding of MYB96 to *ROP* loci. **b** Binding of HDA15 to *ROP* loci in a MYB96-dependent manner. Source data are provided as a Source Data file

We then asked whether the *ROP6*, *ROP10*, and *ROP11* genes are direct regulatory targets of MYB96 and HDA15. To answer this question, we employed 35S:*MYB96-MYC* and 35S:*HDA15-GFP* transgenic plants^43^ and performed chromatin immunoprecipitation (ChIP) using the corresponding antibodies. ChIP-qPCR analysis showed that MYB96 binds to specific regions of the *ROP* promoters (Fig. 5a), and consistently, HDA15 bound to a similar region at each gene promoter (Fig. 5b). The binding regions of MYB96 and HDA15 at the *ROP* chromatins were maintained in transgenic plants expressing native promoter-driven constructs (Supplementary Figure 10).

These observations suggest that MYB96 most likely binds to specific promoter regions and recruits HDA15 that has no DNA-binding specificity. In support, while the *hda15-1* mutation did not influence MYB96 binding to the *ROP* loci (Fig. 5a), binding of HDA15 to the *ROP* promoters was markedly reduced in the *myb96-1* background (Fig. 5b). These results indicate that HDA15 requires MYB96 to associate with the *ROP* promoters and repress expression.

### MYB96–HDA15 complex catalyzes histone deacetylation at *ROP* genes

**Fig. 6:**
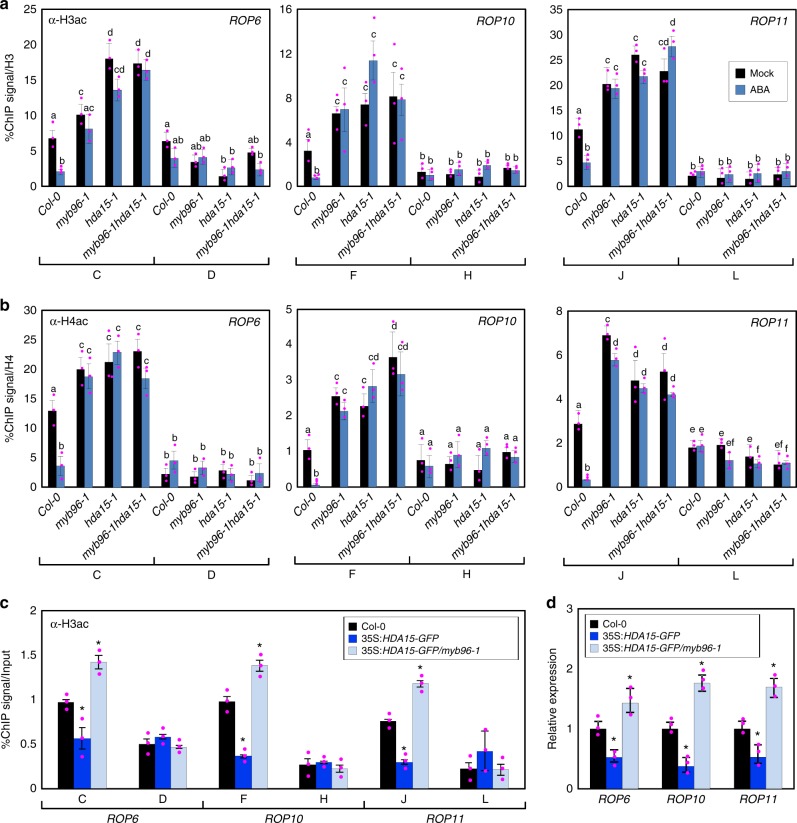
HDA15 and MYB96 co-regulate histone deacetylation at *ROP* loci. **a** Effects of abscisic acid (ABA) on H3ac accumulation at the *ROP* promoters in *myb96-1*, *hda15-1*, and *myb96-1hda15-1* mutants. **b** Effects of ABA on H4ac accumulation at the *ROP* promoters. In **a**, **b**, 2-week-old seedlings grown under long days (LDs) were transferred to MS-liquid medium supplemented with 20 μM ABA and incubated for 24 h. Chromatin immunoprecipitation (ChIP) assays using anti-H3ac and anti-H4ac antibodies were performed. The indicated genomic regions (see Fig. 5) were analyzed by ChIP-quantitative PCR. Three independent biological replicates were averaged, and statistical significance of the measurements was analyzed by analysis of variance (ANOVA) (one-way ANOVA with Fisher’s post hoc test, *P < 0.05). Different letters represent a significant difference at *P* < 0.05. Bars indicate the standard error of the mean. **c** H3ac levels at the *ROP* loci in 35S:*HDA15-GFP* and 35S:*HDA15-GFP*/*myb96-1*. **d** Transcript accumulation of *ROP* genes in wild type, 35S:*HDA15-GFP*, and 35S:*HDA15-GFP/myb96-1*. In **c**, **d**, 2-week-old seedlings grown under LDs were harvested. Statistically significant differences between the wild-type and transgenic plants (Student’s *t* test, *P *<* 0.05) are indicated by asterisks. Source data are provided as a Source Data file

Since HDA15 represses gene expression by promoting H3 and H4 deacetylation^42,43^, we asked whether the MYB96–HDA15 complex catalyzes removal of H3 and H4 acetylation (H3ac and H4ac) at the *ROP* promoters. To this end, ChIP assays using anti-H3ac and anti-H4ac antibodies were performed. In the presence of ABA, acetylation levels of H3 and H4 at the binding regions on the *ROP* promoters were significantly reduced in the wild-type background (Fig. 6a, b). However, ABA repression of H3ac and H4ac accumulation at the *ROP* promoters was compromised in *myb96-1*, *hda15-1*, and *myb96-1hda15-1* mutants (Fig. 6a, b), consistent with the *ROP* transcript levels (Fig. 4c). The histone modifications were most likely propagated into distal regions of the promoters, and farther promoter regions were also modified (Supplementary Figure 11), similar to core-binding regions (Fig. 6a, b). As a negative control, we also analyzed accumulation of H3ac and H4ac at the gene body regions of the *ROP* genes (Fig. 5). As expected, histone acetylation levels were unchanged in *myb96-1*, *hda15-1*, and *myb96-1hda15-1* mutants, regardless of ABA treatment (Fig. 6a, b). These results indicate that MYB96 binds to specific regions of the *ROP* promoters and recruits HDA15 to remove the acetyl groups of H3 and H4 at the promoter regions.

Considering the requirement of MYB96 for HDA15 recruitment (Fig. 5), we wanted to know whether MYB96 is also required for histone deacetylation at the *ROP* promoters. We performed ChIP assays with anti-H3ac antibody using 35S:*HDA15-GFP/myb96-1* plants. While H3ac levels at the *ROP* promoters were significantly reduced in 35S:*HDA15-GFP* transgenic plants compared with wild-type plants even in the absence of ABA, suppression was diminished in 35S:*HDA15-GFP/myb96-1* (Fig. 6c). Consistently, in the absence of ABA, the *ROP* genes were repressed in 35S:*HDA15-GFP* transgenic plants, but this transcriptional repression was restored in 35S:*HDA15-GFP/myb96-1* plants (Fig. 6d). These results indicate that the HDA15 protein binds to the *ROP* promoters and catalyzes histone deacetylation in a MYB96-dependent manner.

### MYB96 represses *ROP* genes in an HDA15-dependent manner

**Fig. 7:**
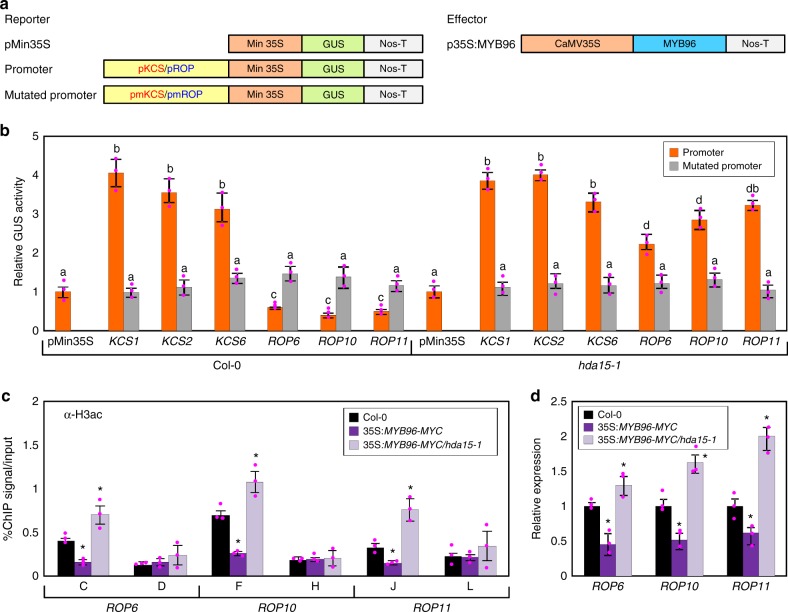
MYB96 represses expression of *ROP* genes in an HDA15-dependent manner. **a** Schematic representation of reporter and effector constructs. The pmKCS and pmROP constructs contain mutated core *cis*-elements (Supplementary Table 3), which impair recognition of R2R3-type MYB DNA-binding domain, on the *KCS* and *ROP* gene promoters, respectively. pMin35S, minimal 35S promoter, CaMV Cauliflower mosaic virus, Nos-T nopaline synthase terminator. **b** Transient expression assays using *Arabidopsis* protoplasts. The recombinant reporter and effector constructs were coexpressed transiently in *Arabidopsis* protoplasts, and GUS activity was determined fluorimetrically. Luciferase gene expression was used to normalize GUS activity. The normalized values in control protoplasts were set to 1 and represented as relative activation. Three independent measurements were averaged. Different letters represent a significant difference at *P* < 0.05 (one-way analysis of variance with Fisher’s post hoc test). Bars indicate the standard error of the mean. **c** H3ac levels in 35S:*MYB96-MYC/hda15-1*. Two-week-old seedlings grown under long days were used to perform chromatin immunoprecipitation (ChIP) using anti-H3ac antibody. The indicated genomic regions (see Fig. 5) were analyzed by ChIP-quantitative PCR. Biological triplicates were averaged, and statistically significant differences between the wild-type and transgenic plants (Student’s *t* test, *P *<* 0.05) are indicated by asterisks. **d** Expression of *ROP* genes in 35S:*MYB96-MYC/hda15-1*. Source data are provided as a Source Data file

We next asked whether MYB96 repression of *ROP* genes also depends on HDA15. To answer this question, we carried out transient expression assays using *Arabidopsis* protoplasts. Reporter constructs, in which the *ROP* and *KETOACYL-COA SYNTHASE* (*KCS*) promoter sequences were fused with the minimal 35S promoter, were coexpressed with an effector construct overexpressing *MYB96* in *Arabidopsis* mesophyll protoplasts isolated from wild-type and *hda15-1* leaves (Fig. 7a). Reporter GUS activity measurement revealed that MYB96 significantly repressed *ROP* promoter activity in the wild-type background, but the repressive function was impaired in the *hda15-1* background (Fig. 7b). The impaired MYB96 function in *hda15-1* was recovered by complementation of *HDA15* (Supplementary Figure 12).

In addition, as a control, we also examined whether HDA15 affects MYB96-dependent ABA-inducible genes. Expression of MYB96 alone was sufficient to activate the *KCS* genes, which are the primary target genes directly activated by MYB96^12^, and the *hda15-1* mutation did not influence MYB96 regulation of ABA-inducible genes (Fig. 7b). Furthermore, coexpression of MYB96 and HDA15 in wild-type protoplasts also did not influence *KCS1* expression (Supplementary Figure 12), suggesting that HDA15 selectively inhibits MYB96-repressed genes.

**Fig. 8:**
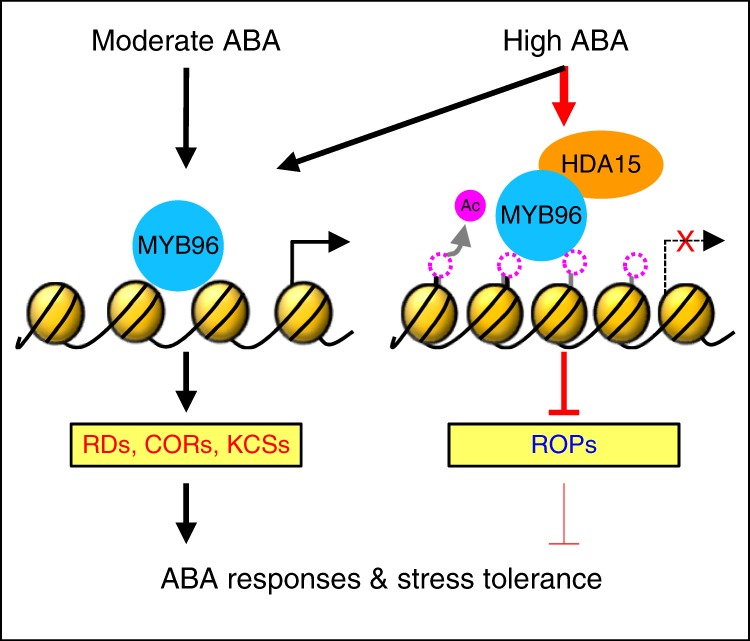
Proposed working model. In the presence of abscisic acid (ABA), MYB96 alone is sufficient to promote expression of ABA-induced genes, including *KCS* genes. However, MYB96 requires HDA15 to repress the ABA-repressed genes *ROP6*, *ROP10*, and *ROP11*. MYB96 interacts with HDA15 especially at high concentrations of ABA. MYB96 and HDA15 bind to the *ROP* promoters and repress expression by promoting H3 and H4 deacetylation. MYB96 confers strong ABA responses by simultaneously activating positive regulators and suppressing negative regulators of ABA signaling

We also measured H3ac accumulation at the *ROP* promoters in 35S:*MYB96-MYC* and 35S:*MYB96-MYC/hda15-1* plants. As expected, H3ac levels at the *ROP* promoters were reduced in 35S:*MYB96-MYC* even in the absence of ABA, but reduction in H3ac levels was impaired in the *hda15-1* background (Fig. 7c). Transcript accumulation of the *ROP* genes further supported these observations. Reduction of the *ROP* genes in 35S:*MYB96-MYC* was restored in 35S:*MYB96-MYC*/*hda15-1* (Fig. 7d). These observations indicate that the role of MYB96 in *ROP* repression depends on HDA15. Overall, the MYB96–HDA15 complex represses the *ROP* genes, which are negative regulators of ABA signaling, through histone deacetylation to strongly promote ABA responses (Fig. 8).

## Discussion

Transcriptional responses to environmental challenges are usually linked to chromatin regulation^44,45^. Accumulating evidence has shown that genome-wide chromatin modification/remodeling accompanies water-deficit stress^46–49^. For instance, global changes in histone variant H2A.Z deposition occur especially in stress-responsive genes, and the *arp6* mutant exhibits higher sensitivity to osmotic stress with hyperactivation of stress-responsive genes^50^.

Consistently, a significant number of chromatin modifiers/remodelers are differentially expressed in response to ABA and/or drought stress to confer appropriate responses to environmental disadvantages^50–54^. As a supporting evidence, the *Arabidopsis* SWI/SNF chromatin-remodeling ATPase BRAHMA (BRM) represses ABA responses by directly promoting occupancy of the +1 nucleosome at the transcription start site of the *ABI5* locus in the absence of ABA^55^. *ABI5* expression is derepressed in *brm* mutants with reduction of the +1 nucleosome occupancy^55^, and consistently, *brm* mutants exhibit ABA hypersensitivity^55^. Moreover, BRM interacts with SnRK2s and clade A PP2Cs, and BRM function is further shaped by its phosphorylation state^56^. When ABA levels increase, SnRK2s are activated and clade A PP2Cs are inhibited by PYR/PYL ABA receptors. ABA-activated SnRK2s phosphorylate BRM, which leads to inhibition of BRM activity and thereby *ABI5* induction. Under normal growth conditions with low ABA levels, dephosphorylation of BRM by PP2Cs restores BRM activity and represses *ABI5* expression^56^.

Several histone modifiers are also involved in abiotic stress responses. Two evolutionarily conserved *Arabidopsis* Trithorax Group proteins (TrxGs), ARABIDOPSIS TRITHORAX 4 (ATX4) and ATX5, directly bind to the *ABA-HYPERSENSITIVE GERMINATION 3* (*AHG3*) locus, which acts as a negative regulator in ABA signaling, and regulate H3K4me3 deposition to activate expression^57^. Accordingly, the *atx4* and *atx5* single and the *atx4 atx5* double mutants exhibit higher drought tolerance and ABA-hypersensitive phenotypes^57^. The RPD3-type histone deacetylases HDA6 and HDA19 also regulate the expression of ABA and abiotic stress-responsive genes by forming huge transcriptional repressor complexes, together with HISTONE DEACETYLATION COMPLEX 1 (HDC1), MULTICOPY SUPRESSOR OF IRA 1 (MSI1), ETHYLENE RESPONSE FACTOR 7 (ERF7), HISTONE DEACETYLASE 2 C (HD2C), and SWI-INDEPENDENT 3 (SIN3) in *Arabidopsis*^19,58–61^. These complexes usually repress expression of ABA-related genes by promoting histone deacetylation at cognate loci. Mutations of the components result in hyperactivation of ABA-responsive genes and thus hypersensitivity to ABA and salt stress and increased tolerance of osmotic stress^19,58–60,62,63^.

In this study, we found that HDA15 acts as a positive regulator of ABA signaling by repressing *ROP* genes, which are pivotal repressors of early ABA signaling^26,27,29,41^. Binding selectivity of HDA15 to the *ROP* loci is defined by the MYB96 transcription factor. In the presence of ABA, the MYB96–HDA15 complex co-binds to the *ROP* promoters and removes acetyl groups of histone H3 and H4 to stimulate ABA responses. Consistently, *hda15* and *myb96* mutants exhibit reduced ABA sensitivity, whereas transgenic plants overexpressing *HDA15* and *MYB96* are hypersensitive to ABA. Overall, ABA signaling is shaped by multiple layers of regulation^64^, and epigenetic mechanisms are a key regulatory scheme in plant stress responses.

MYB96 regulates early signaling components of ABA pathways, including the *ROP* genes. Consistently, MYB96 has a strong impact on plant tolerance to abiotic stresses^10,12–14,31^ and regulates a variety of ABA responses, including lateral root development, stomatal movement, hormone biosynthesis, and cuticular wax biosynthesis^10,12–14,31^. Like many plant transcription factors, MYB96 functions as a transcriptional repressor for some promoters but an activator for others. Our preliminary genome-wide identification of direct binding sites of MYB96 provides support that MYB96 binds to promoters of both ABA-repressed and ABA-induced genes. MYB96 transcriptional activity would be determined in part by trans-factors, such as histone-modifying enzymes. In this study, we found that, while MYB96 alone is sufficient to activate ABA-inducible genes such as *RD* and *KCS* genes, MYB96 requires HDA15 to inhibit the expression of ABA-repressible genes. In particular, the MYB96–HDA15 complex selectively represses the *ROP* genes, which are negative regulators of ABA signaling.

Given the synergistic interactions between MYB96 and HDA15, the HDA15 protein most likely has regulatory targets non-overlapping with MYB96 in the control of ABA responses, although HDA15 target genes are elusive so far. HDA15 alone can confer ABA sensitivity independently of MYB96. Probably, each individual member deals with mild ABA responses, but the physical interaction between MYB96 and HDA15 enables to induce strong and robust ABA responses by regulating upstream core components.

One remaining question is how HDA15 selectively represses the *ROP* genes without affecting MYB96-activated genes. Physical interactions with additional ABA signaling components or trans-factors might be involved. Moreover, the strength of MYB96 activation may also be involved. MYB96 activation above the low threshold allows it to mainly act as a transcriptional activator to promote genes contributing to stress tolerance in plants. At a high level of MYB96 activation, a significant portion of MYB96 proteins above a high threshold may get a chance to interact with HDA15, which is also significantly activated under harsh stress conditions, to additionally repress negative regulators of ABA signaling, further enhancing ABA responses. In support, MYB96–HDA15 interactions are profoundly enhanced at high concentrations of ABA. Taken together, MYB96 is most likely a master regulator of ABA signaling. This transcription factor not only activates positive regulators of ABA signaling but also represses repressors of ABA signaling, ensuring full activation of ABA responses.

HDA15 function is shaped by light conditions. HDA15 interacts with PHYTOCHROME INTERACTING FACTOR 1 (PIF1) and PIF3^42,43^, which act as negative regulators in light responses and repress light-mediated cotyledon development, chlorophyll biosynthesis, and hormonal signaling in the dark^65–70^. The HDA15–PIF complexes co-bind to the target genes and reduce histone H3 acetylation levels, repressing expression in the dark^42,43^. Upon light exposure, active forms of phytochromes translocate into the nucleus and induce phosphorylation-induced degradation of PIFs, leading to the dissociation of HDA15 from chromatin and activation of the light-responsive genes^42,43^.

Active roles of HDA15 in light have also been demonstrated. NF-YC1, NF-YC3, NF-YC4, and NF-YC9 interact with HDA15 in the light, and they co-target the promoters of a set of hypocotyl elongation-related genes, such as *INDOLE-3-ACETIC ACID INDUCIBLE 19* (*IAA19*) and *XYLOGLUCAN ENDOTRANSGLUCOSYLASE/HYDROLASE 17* (*XTH17*), to repress H4 acetylation levels at the associated regions. As a consequence, hypocotyl elongation is suppressed in light^71,72^. However, in the dark, HDA15 dissociates from the target genes, resulting in increased H4ac levels and thereby etiolated growth^71^.

The interactions between HDA15 and MYB96 might also be dependent on light conditions. Consistent with the fact that efficient ABA responses are required during the daytime, when plants are usually subjected to water deficit, many ABA signaling mediators, including MYB96, are activated in light conditions^30,73,74^. MYB96–HDA15 interaction might be relevant during the day to facilitate activation of drought tolerance by repressing negative regulators of ABA signaling. Plants need to ensure a perfect balance between water loss and photosynthesis^75,76^. The point of crosstalk between light and stress responses not only maximizes metabolic efficiency and growth but also establishes an efficient way to increase stress tolerance under environmentally unfavorable conditions.

## Methods

### Plant materials and growth conditions

*Arabidopsis thaliana* (Columbia-0 ecotype) was used for all experiments unless otherwise specified. Plants were grown under long-day conditions (LDs; 16-h light/8-h dark cycles) with cool white fluorescent light (120 μmol photons/m/s) at 22–23 °C. *myb96-ox*, *myb96-1*, *hda15-1*, 35S:*MYB96-MYC*, and 35S:*HDA15-GFP* were previously reported^10,12,43^. To produce transgenic plants overexpressing the *MYB96* gene, full-length cDNA was subcloned into the modified binary pBA002 vector under the control of the CaMV 35S promoter^77^. *Agrobacterium tumefaciens*-mediated *Arabidopsis* transformation was then performed. *myb96-1hda15-1*, *pMYB96:MYB96-MYC x pHDA15:HDA15-GFP*, 35S:*MYB96-MYC*/*hda15-1*, and 35S:*HDA15-GFP*/*myb96-1* plants were obtained by genetic crosses.

### Seed germination assays

All seeds were harvested at the same time and dried at least 1 month for each genotype. For seed germination assays, seeds for each genotype (>40 seeds per replicate) were sterilized and stratified for 2 days at 4 °C in darkness. Stratified seeds were transferred to a culture room at 23 °C under LDs and germinated on MS medium (half-strength MS salts, 0.05% MES, pH 5.7 and 1% agar) supplemented with various concentrations of ABA. To measure seed germination percentage, radicle emergence from the seed coat and endosperm was used as a phenotypic marker and counted at regular intervals. Each germination assay was performed in biological triplicates.

### ABA treatment and drought

For ABA treatment, 2-week-old seedlings grown under LDs were transferred to half-strength MS-liquid medium supplemented with 20 μM (+)-*cis,trans*-ABA (L06278) (Alfa Aesar, Ward Hill, MA, USA). Drought stress was induced in 2-week-old plants grown in soil under LD conditions by halting watering. To prevent direct air-drying of seedlings, small pores were made in the plastic cover 7 days after the start of drought conditions, and the cover was removed 7 days later. Survival rates were measured for each group of plants. Three biologically independent measurements of at least 30 plants were averaged.

### RT-qPCR analysis

Total RNA samples were extracted from whole seedlings using TRI reagent (TAKARA Bio, Singa, Japan) according to the manufacturer’s recommendations. Total RNA samples were pretreated with an RNAse-free DNAse to eliminate genomic DNA contamination. The first-strand cDNA was synthesized from 2 μg of total RNA by reverse transcription using Moloney Murine Leukemia Virus reverse transcriptase (Dr. Protein, Seoul, Korea) with oligo(dT18).

RT-qPCR experiments were performed on the Step-One Plus Real-Time PCR System (Applied Biosystems). The PCR primers used are listed in Supplementary Table 1. The comparative *C*_T_ method was used to determine the relative gene expression with the expression of the *EUKARYOTIC TRANSLATION INITIATION FACTOR 4A1* (*eIF4A*) gene (At3g13920) as an internal control. All RT-qPCR reactions were performed using three independent replicate samples. The specificity of RT-qPCR results was determined by melt curve analysis of the amplified products using the standard method.

### Y2H assays

For Y2H assays, the expression vectors and yeast strains used were supplied by the BD Matchmaker system (Clontech, Mountain View, CA, USA). The yeast strain AH109 harboring the *LacZ* and *His* reporter genes was used. PCR products were subcloned into the pGBKT7 (for GAL4 BD fusion) and pGADT7 (for GAL4 AD fusion) vectors. The recombinant constructs were cotransformed into AH109 cells, and the transformed cells were grown on SD/-Leu/-Trp medium and SD/-Leu/-Trp/-His/-Ade medium. The interaction strength was quantified by measuring β-galactosidase (β-Gal) activity using *o*-nitrophenyl-β-d-galactopyranoside (ONPG) as a substrate.

### BiFC assays

The full-size *MYB96* and *HDA15* coding sequence were fused in-frame to the 5’ end of a gene sequence encoding the C-terminal half of EYFP in the pSATN-cEYFP-C1 vector (E3082) or the N-terminal half of EYFP in the pSATN-nEYFP-C1 vector (E3081)^78^. The IDD14-RFP construct was used as a nucleus localization marker^79^. The recombinant constructs were co-transfected into *Arabidopsis* mesophyll protoplasts by the PEG-calcium transfection method. Transformed protoplasts were incubated at 23 °C for 12–16 h in darkness. Emitted fluorescence was monitored by Zeiss LSM510 confocal microscope (Carl Zeiss, Jena, Germany).

### ChIP assays

Harvested plant materials were cross-linked with 1% formaldehyde, ground in liquid nitrogen, and solubilized in NLB buffer (50 mM HEPES, pH 7.5, 150 mM NaCl, 0.5 mM EDTA, 1% Triton X-100, 0.1% sodium deoxycholate, 0.1% sodium dodecyl sulfate (SDS), 1 μg/mL pepstatin A, 1 μg/mL aprotinin, 1 μg/mL leupeptin, and 1 mM phenylmethylsulfonyl fluoride). Chromatin was fragmented on ice with a probe sonicator to obtain approximately 200–800-bp fragments. After sonication, the suspension was centrifuged twice for 20 min at 13,200 × *g*. The extract was precleared with 100 μL of salmon sperm DNA/protein A agarose beads (16–157, Millipore, Billerica, USA) for 1 h. The precleared supernatant was incubated overnight with 10 μL of corresponding antibodies, including anti-MYC (05-724, Millipore; 1:500 dilution), anti-H3ac (06-599, Millipore; 1:500 dilution), anti-H4ac (06-866, Millipore; 1:500 dilution), anti-GFP (ab290, Abcam, Cambridge, USA; 1:500 dilution), or no antibody (control). Then the supernatant was mixed with 100 μL of salmon sperm DNA/protein A agarose beads and incubated for 3 h on a rotating wheel. The beads were washed with 10 mL of NLB buffer, 1 mL of low-salt buffer (20 mM Tris-Cl, pH 8, 150 mM NaCl, 2 mM EDTA, 1% Triton X-100, and 0.1% SDS), 1 mL of high-salt buffer (20 mM Tris-Cl, pH 8, 500 mM NaCl, 2 mM EDTA, 1% Triton X-100, and 0.1% SDS), and 1 mL of LiCl buffer (10 mM Tris-Cl, pH 8, 0.25 M LiCl, 1 mM EDTA, 1% sodium deoxycholate, and 1% Nonidet P-40). DNA was purified using phenol/chloroform/isoamyl alcohol and sodium acetate (pH 5.2). The levels of precipitated DNA fragments were quantified by qPCR using specific primer sets (Supplementary Table 2). Values were normalized according to input DNA levels. Values for control plants were set to 1 after normalization against *eIF4a* for qPCR analysis.

### Transient expression assays

For transient expression assays using *Arabidopsis* protoplasts, reporter and effector plasmids were constructed. The reporter plasmid (modified pCAMBIA1305 vector) contains a minimal 35S promoter sequence and the GUS-coding gene^80^. The core *cis*-elements on the *KCS* and *ROP* promoters were inserted into the reporter plasmid. pmKCS and pmROP mutant constructs were generated with mutations within core *cis*-elements (Supplementary Table 3). To construct effector plasmids, *MYB96* and *HDA15* cDNAs were inserted into the effector vector pBA002 containing the CaMV 35S promoter.

Fifth leaves were cut into 0.5-mm strips with a fresh razor blade and digested with gentle shaking in an enzyme solution containing 1% (w/v) cellulose RS, 0.1% (w/v) macerozyme R10 (Karlan Biochemicals, Cottonwood AZ), 0.6 M mannitol, 10 mM MES (pH 5.7), 1 mM CaCl_2_, 1 mM MgCl_2_, 10 mM β-mercaptoethanol, and 0.1% bovine serum albumin (w/v) for 3 h at room temperature. The enzyme solution was sieved through a 70-μm nylon mesh (Carolina Biologicals, Burlington, NC) and spun at 45 × *g* for 5 min. The pellet was washed twice and re-suspended in wash solution (0.6 M mannitol, 4 mM MES, pH 5.7). Recombinant reporter and effector plasmids were cotransformed into *Arabidopsis* protoplasts by polyethylene glycol-mediated transformation. After 16-h incubation in the dark at 23 °C, transformed protoplasts were processed for quantifying β-glucuronidase (GUS) activity. A CaMV 35S promoter-luciferase construct was also cotransformed as an internal control. The luciferase assay was performed using the Luciferase Assay System Kit (Promega, Madison, WI).

### Co-IP assays

*A. tumefaciens* cells containing 35S:*MYB96-MYC* and 35S:*HDA15-GFP* constructs were infiltrated to 3-week-old *N. benthamiana* leaves. Tobacco leaves were homogenized in liquid nitrogen, and total proteins were extracted in protein extraction buffer (25 mM Tris-HCl, pH 7.5, 150 mM NaCl, 5% glycerol, 0.05% Nonidet P-40, 2.5 mM EDTA, 1 mM phenylmethylsulfonyl fluoride, and 1× complete cocktail of protease inhibitors). After protein extraction, 5% of the extracts were used as input control. The protein extracts were mixed with anti-MYC antibodies (05-724, Millipore, Billerica, MA, USA; 1:500 dilution) coupled to Protein-A sepharose beads (Sigma-Aldrich, St Louis, MO, USA) and incubated for 46 h at 4 °C. The precipitated samples were washed at least four times with the protein extraction buffer and then eluted by 1× SDS–polyacrylamide gel electrophoresis (PAGE) loading buffer to subject to SDS–PAGE with anti-MYC (1:2000 dilution; Millipore) or anti-GFP antibodies (1:500 dilution; sc-9996, Santa Cruz Biotech., Dallas, TX, USA). Similar protocols were used for Co-IP assays for *Arabidopsis* plants.

### Reporting Summary

Further information on experimental design is available in the Nature Research Reporting Summary linked to this article.

## Supplementary information

Supplementary Information

Peer Review

Reporting Summary

Source Data

## Data Availability

Data supporting the findings of this work are available within the paper and its Supplementary Information files. A reporting summary for this article is available as a Supplementary Information file. The source data underlying Fig. 1a, c–e, 2a–g, 3a–d, 4a–c, 5a, b, 6a–d, and 7b–d, as well as Supplementary Figures 1–12 are provided as a Source Data file. The datasets generated and analyzed during the current study are available from the corresponding author on reasonable request.
