# Supplementary material for: MYB96 recruits the HDA15 protein to suppress negative regulators of ABA signaling in Arabidopsis
Source: Nat Commun. 2019 Apr 12;10:1713. doi: 10.1038/s41467-019-09417-1 (PMC6461653; doi:10.1038/s41467-019-09417-1)
Supplement: Supplementary file 1 — Supplementary Information [file 41467_2019_9417_MOESM1_ESM.pdf]

MYB96 recruits the HDA15 protein to suppress negative regulators of ABA signaling in *Arabidopsis*.

Lee and Seo

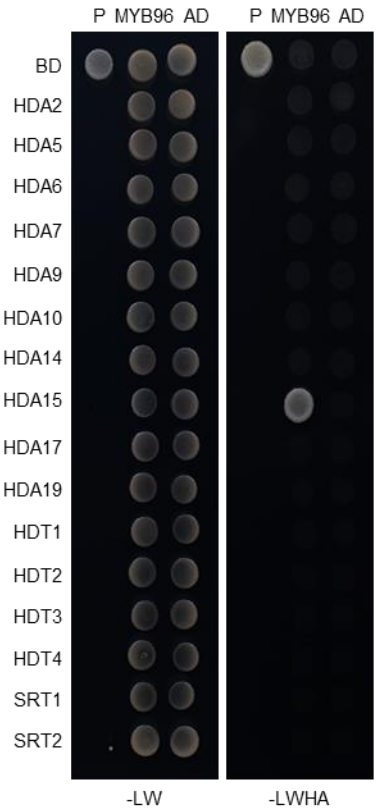

Supplementary Figure 1. Yeast-two-hybrid (Y2H) assays.
Y2H assays were performed with HDAC proteins fused with the DNA binding domain (BD) of GAL4 and MYB96 fused with the transcriptional activation domain (AD) of GAL4 for analysis of their interactions. Interactions were examined by cell growth on selective media. -LWHA indicates Leu, Trp, His, and Ade drop-out plates. -LW indicates Leu and Trp drop-out plates. GAL4 was used as a positive control (P). Source data are provided as a Source Data file.

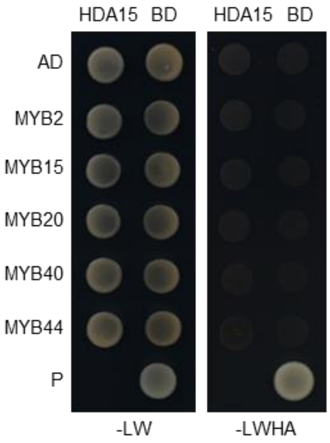

Supplementary Figure 2. Interactions of HDA15 with ABA-responsive MYB transcription factors. The GAL4 BD-HDA15 fusion was coexpressed with MYB transcription factors fused with GAL4 AD in yeast cells. Interactions were examined by cell growth on selective media. -LWHA indicates Leu, Trp, His, and Ade drop-out plates. -LW indicates Leu and Trp drop-out plates. GAL4 was used as a positive control (P). Source data are provided as a Source Data file.

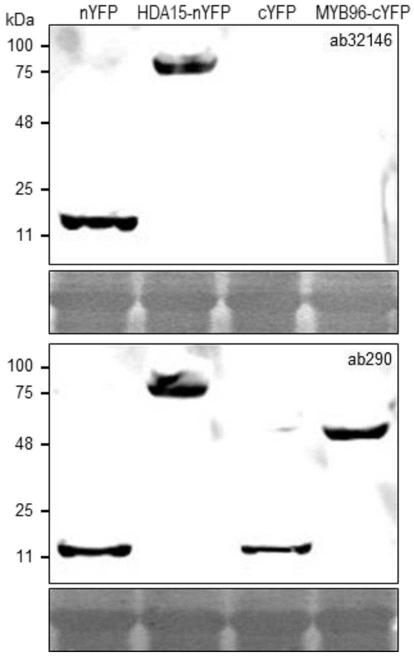

Supplementary Figure 3. Validation of protein expression in transfected *Arabidopsis* protoplasts. Partial YFP fusion constructs containing either HDA15 or MYB96 were transiently expressed in *Arabidopsis* protoplasts. Proteins were immunologically detected using ab32146 (for N-terminal YFP fragment) and ab290 (for full-length YFP protein) antibodies. Note that full-sized proteins were expressed, and similar protein levels were detected in each transfected sample. Source data are provided as a Source Data file.

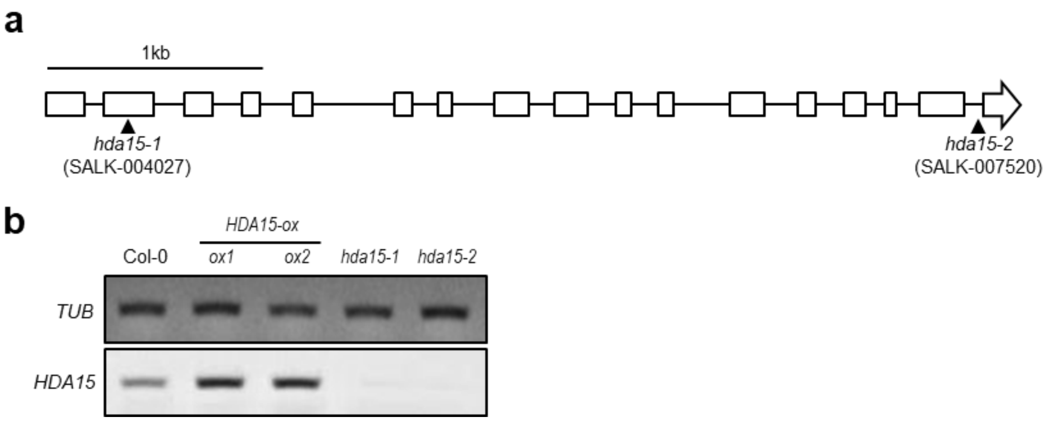

Supplementary Figure 4. Transcript accumulation of *HDA15* in *hda15* mutant and *HDA15-ox* transgenic plants.

(a) Mapping of the T-DNA insertion sites of *hda15*-deficient mutants. White arrows indicate exons. Black arrowheads indicate T-DNA insertion sites.

(b) Transcript accumulation of *HDA15*. Two-weeks-old seedlings grown under long-day conditions (LDs) were harvested for total RNA isolation. Transcript accumulation was analyzed by semi-quantitative RT-PCR. The *TUBULIN BETA CHAIN 2* (*TUB*) gene (At5g62690) was used as an internal control.

Source data are provided as a Source Data file.

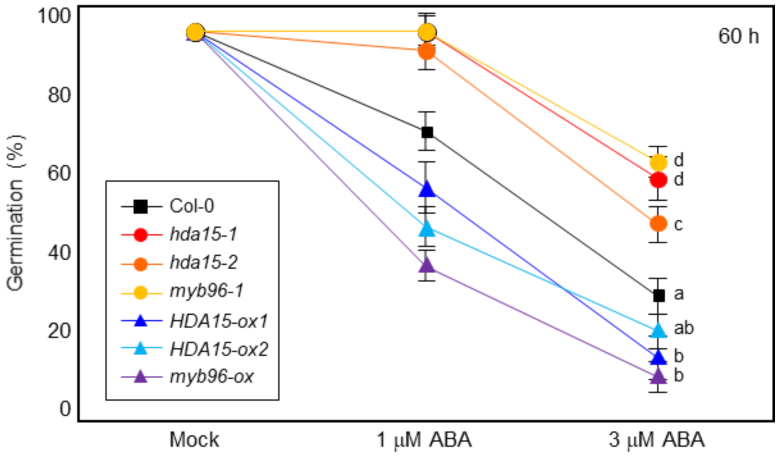

Supplementary Figure 5. ABA dose-response in seed germination.

Seeds were germinated on an increasing concentration of ABA. Germination percentages were scored 60 hours (h) after cold stratification. Biological triplicates were averaged. Different letters represent a significant difference at $P < 0.05$ (one-way ANOVA with Fisher's *post hoc* test). Bars indicate the standard deviation of the mean. Source data are provided as a Source Data file.

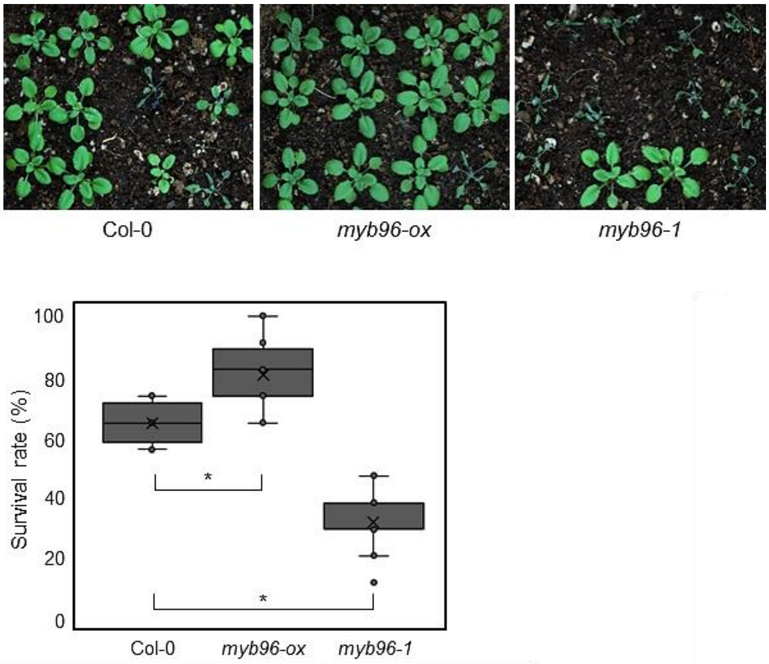

Supplementary Figure 6. Drought tolerance of *myb96-ox* and *myb96-1* plants.

Two-week-old plants were treated for drought stress by stopping watering for an additional 2 weeks. Three independent biological replicates were averaged, and statistically significant differences between the wild-type and transgenic or mutant plants are indicated by asterisks (Student's *t*-test, $*P < 0.05$). Source data are provided as a Source Data file.

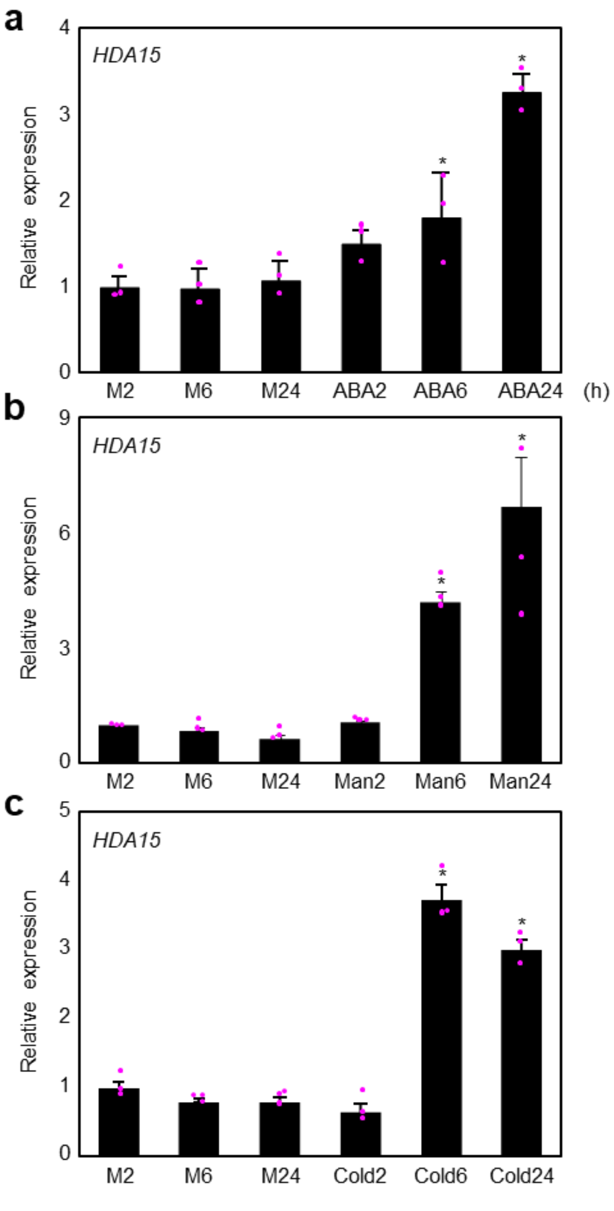

Supplementary Figure 7. Effects of ABA and osmotic and cold stress on *HDA15* expression.

Two-week-old seedlings grown at 23°C under LDs were transferred to MS-liquid medium supplemented with 20 μM ABA (**a**) or 150 mM mannitol (Man) (**b**) or exposed to 4°C (**c**) and then incubated for the indicated time period (h). Three independent biological replicates were averaged, and statistical significance of the measurements was analyzed by a Student's *t*-test (**P* < 0.05). Bars indicate the standard error of the mean. M, mock. Source data are provided as a Source Data file.

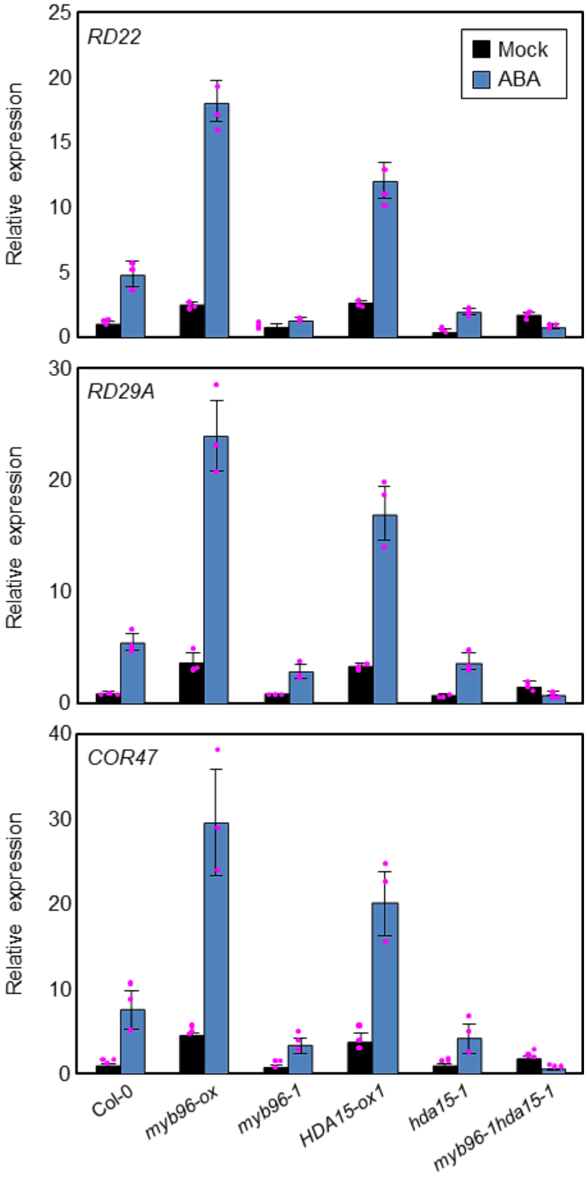

Supplementary Figure 8. HDA15 regulation of ABA induction of ABA marker genes at early seedlings.

Early seedlings at 2-day after germination were transferred to MS-liquid medium supplemented with or without 20 μM ABA and incubated for 24 h. Transcript accumulation was analyzed by RT-qPCR. Three independent biological replicates were averaged. Bars indicate the standard error of the mean. Source data are provided as a Source Data file.

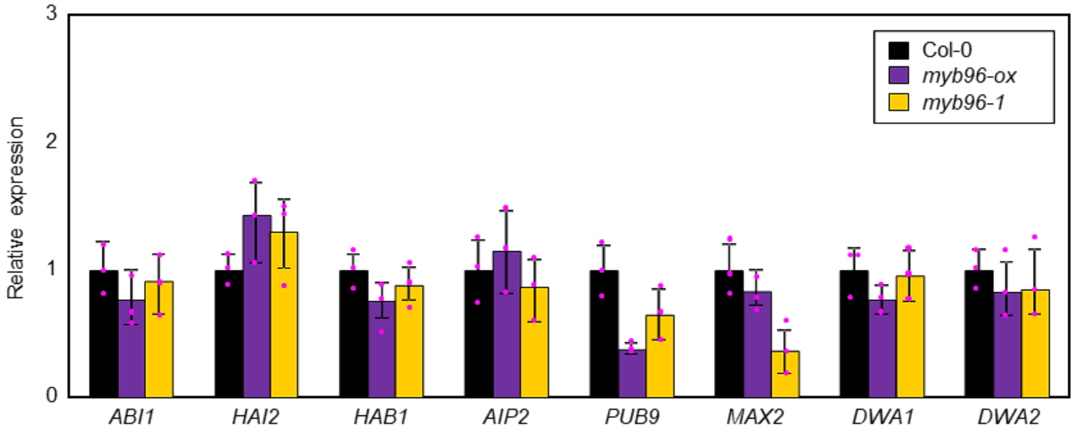

Supplementary Figure 9. Expression of genes encoding negative regulators of ABA signaling in *myb96-ox* and *myb96-1* mutant plants.

Two-week-old plants grown under LD conditions were used to analyze transcript accumulation. The *eIF4a* gene was used as an internal control. Biological triplicates were averaged. Bars indicate the standard error of the mean. Source data are provided as a Source Data file.

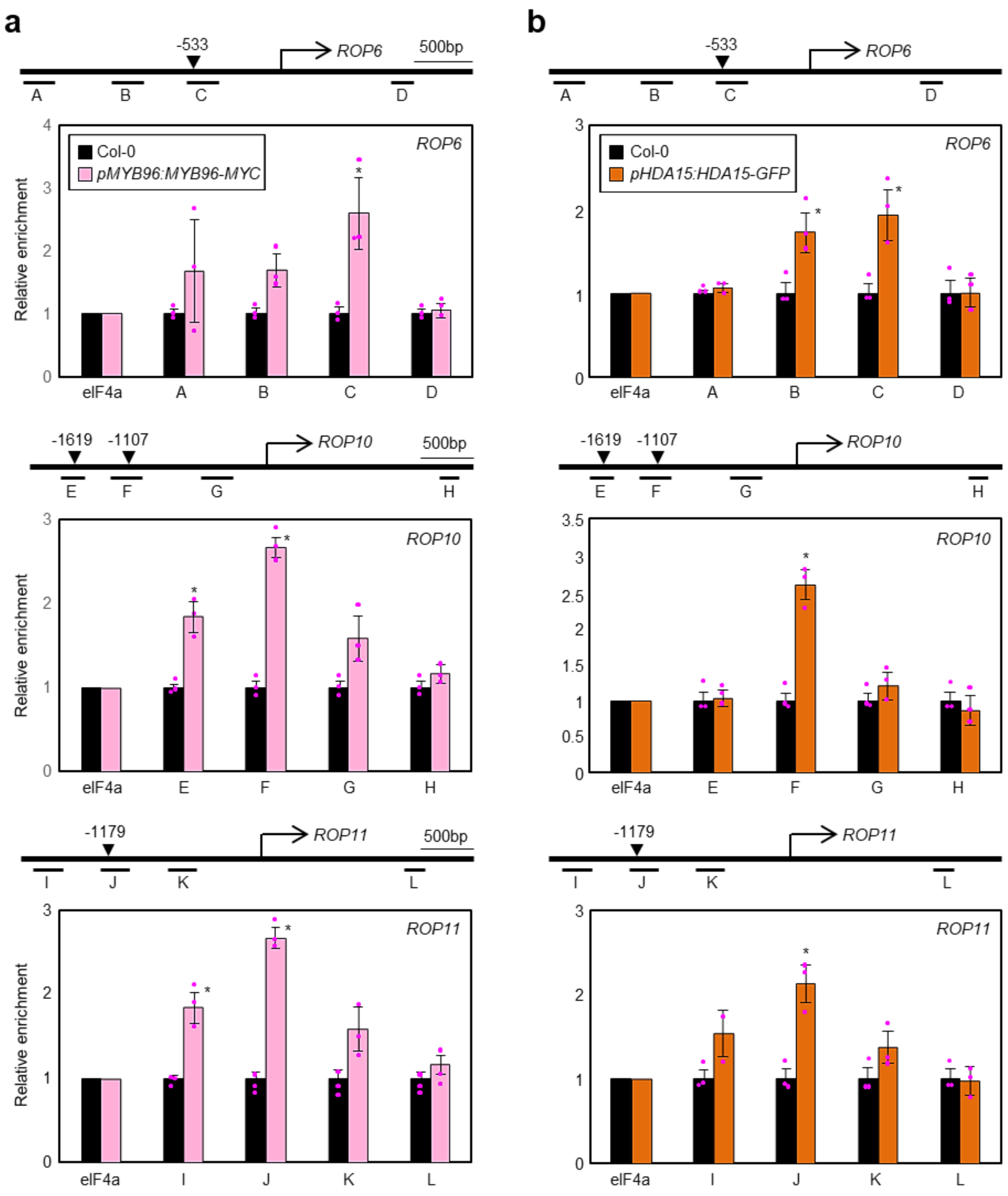

Supplementary Figure 10. Binding of MYB96 and HDA15 to the *ROP* promoters.

Putative MYB binding sites are indicated by arrowheads. Black underbars indicate the regions of PCR amplification after chromatin immunoprecipitation (ChIP). ChIP-qPCR assays were conducted to examine enrichment of MYB96 (**a**) and HDA15 (**b**) at the *ROP* loci. Three independent biological replicates were averaged, and statistical significance of the measurements was analyzed by a Student's *t*-test (**P* < 0.05). Bars indicate standard error of the mean. Source data are provided as a Source Data file.

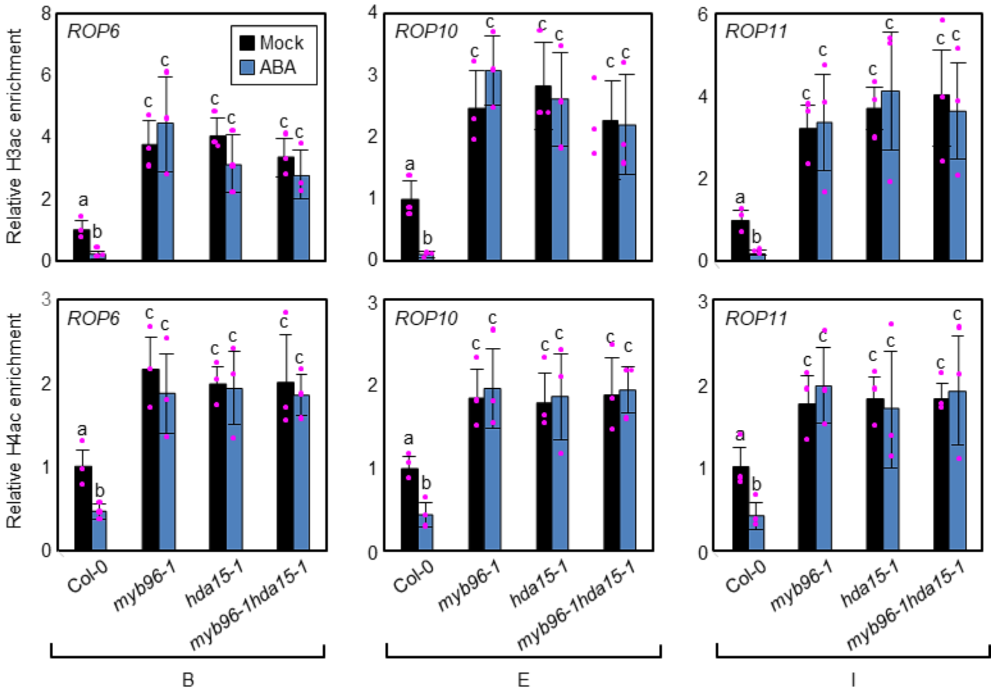

Supplementary Figure 11. Effects of ABA on H3ac and H4ac accumulation at the *ROP* promoters. Two-weeks-old seedlings grown under LDs were transferred to MS-liquid medium supplemented with 20 μM ABA and incubated for 24 h. ChIP assays using anti-H3ac and anti-H4ac antibodies were performed. The indicated genomic regions (see Fig. 5) were analyzed by ChIP-qPCR. Three independent biological replicates were averaged, and statistical significance of the measurements was analyzed by ANOVA (one-way analysis of variance with Fisher's *post hoc* test, $^{*}P < 0.05$). Bars indicate the standard error of the mean. Source data are provided as a Source Data file.

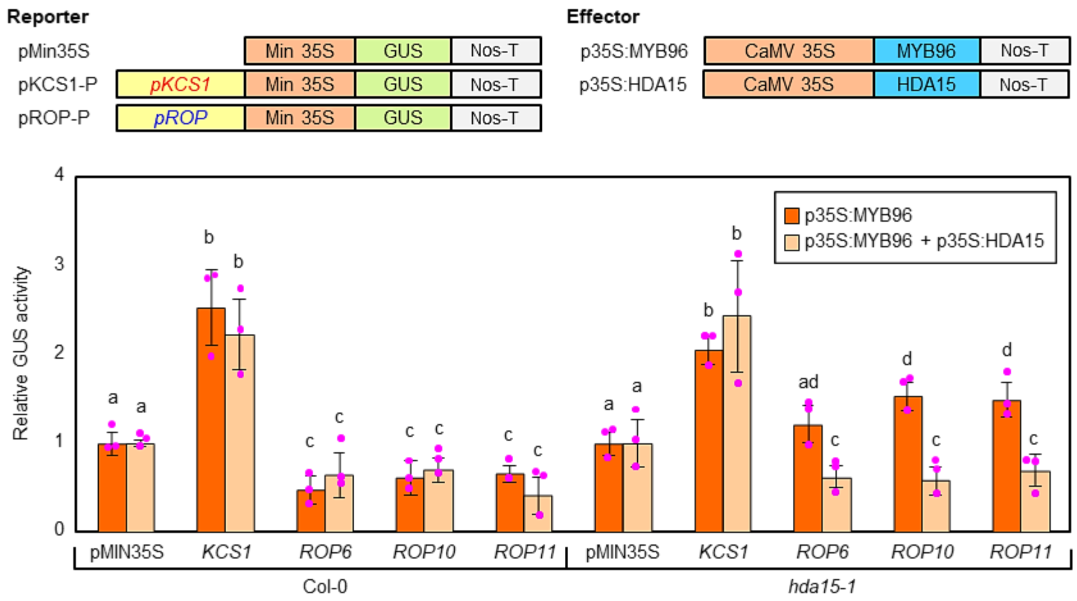

Supplementary Figure 12. Transient expression assays using *Arabidopsis* protoplasts.

The recombinant reporter and effector constructs were coexpressed transiently in *Arabidopsis* protoplasts, and GUS activity was determined fluorimetrically. Luciferase gene expression was used to normalize GUS activity. The normalized values in control protoplasts were set to 1 and represented as relative activation. Three independent measurements were averaged. Different letters represent a significant difference at $P < 0.05$ (one-way ANOVA with Fisher's *post hoc* test). Bars indicate the standard error of the mean. Source data are provided as a Source Data file.

Supplementary Table 1. Primers used in this study.

Primer	Usage	Sequence
eIF4a-F	RT-qPCR	5'-TGACCACACAGTCTCTGCAA
eIF4a-R	RT-qPCR	5'-ACCAGGGAGACTTGTTGGAC
MYB96-F	RT-qPCR	5'-TGCAGTCTCGGAAGAAGGTG
MYB96-R	RT-qPCR	5'-CATCTCGTGGCTTTGCTCAT
HDA15-F	RT-qPCR	5'-ATTTGATGCGGCTAGAGGAG
HDA15-R	RT-qPCR	5'-TTTCCCACCACACAGATCAC
RD22-F	RT-qPCR	5'-AGGAGCAAACCCTTTCGTGT
RD22-R	RT-qPCR	5'-CGTTTCAACGTCTCCGAAAA
RD29A-F	RT-qPCR	5'-GTTACTGATCCCACCAAAGAAGA
RD29A-R	RT-qPCR	5'-GGAGACTCATCAGTCACTTCCA
COR47-F	RT-qPCR	5'-GGACACCACGACAAGACAGC
COR47-R	RT-qPCR	5'-CGTGACAGCTGGTGAATCCT
ROP1-F	RT-qPCR	5'-GCAGGGCAAGAGGATTACAA
ROP1-R	RT-qPCR	5'-TTTGGAACTGCATTCGATGT
ROP2-F	RT-qPCR	5'-TTTGAGTTACCGTGGTGCTG
ROP2-R	RT-qPCR	5'-GCATCAAACACTGCCTTCAC
ROP3-F	RT-qPCR	5'-TCCAAGAAGTGGATCCCAGA
ROP3-R	RT-qPCR	5'-AATGGAGCAGGCTTTTTGTG
ROP4-F	RT-qPCR	5'-GGTGCCGATGTCTTCATTCT
ROP4-R	RT-qPCR	5'-CACTTTTATGGCTGCGTCAA
ROP5-F	RT-qPCR	5'-GGTGATGGAGCTGTTGGTAAA
ROP5-R	RT-qPCR	5'-TGGGACACCAGGAGCATAGT
ROP6-F	RT-qPCR	5'-GCCAATGTGATTGTTGATGG
ROP6-R	RT-qPCR	5'-TTCACCCTGAGCGGTAGAGA
ROP7-F	RT-qPCR	5'-ACTGTCGGAGATGGAGCAGT
ROP7-R	RT-qPCR	5'-GCCAGGAGCATAATGTTTCA
ROP8-F	RT-qPCR	5'-CGTTCCCCACTGATTATGTTC
ROP8-R	RT-qPCR	5'-TTGGGAACTGCATGTTGTCT
ROP9-F	RT-qPCR	5'-GCCTGAACTTCGTCGGTTT
ROP9-R	RT-qPCR	5'-ATACTCGCAATGGAGCAACC
ROP10-F	RT-qPCR	5'-GGCATCACTGTGAACTTAGGC
ROP10-R	RT-qPCR	5'-GATATGCTTGCGGAGTTCCT
ROP11-F	RT-qPCR	5'-TTGTTGAAGGCACCACTGTC
ROP11-R	RT-qPCR	5'-TACGCAACTCCTCTCCCTGT
ABI1-F	RT-qPCR	5'-CGTCTCACATCTTCGTCGCT
ABI1-R	RT-qPCR	5'-TCAATCCTCGCAGCTTCATC
HAI2-F	RT-qPCR	5'-TTGGGGTGAAACTGTGATGA
HAI2-R	RT-qPCR	5'-CGGACGATCAGGCTTATGAT
HAB1-F	RT-qPCR	5'-TGTTGCGTCTGAGACCGTAG
HAB1-R	RT-qPCR	5'-ATGGCGAGAACACCAAAAAC
AIP2-F	RT-qPCR	5'-GCACACATTTCACCCTCCTT
AIP2-R	RT-qPCR	5'-CCTCTCTTCTTCGGCCTCTT
PUB9-F	RT-qPCR	5'- TCGAATGGGTTGTACGTTGA
PUB9-R	RT-qPCR	5'- CACTTTGTCCTGTCGCTGAA
MAX2-F	RT-qPCR	5'- GTGCCTGACTTTGAGGAAGC
MAX2-R	RT-qPCR	5'- AATCGGCTACACGAACCAAC
DWA1-F	RT-qPCR	5'- GGCTGAACCTGAGAGAGTGG
DWA1-R	RT-qPCR	5'- TCAAGCAATGGCTTCAGATG
DWA2-F	RT-qPCR	5'- GAGTCAGCTGGTATGCGTCA
DWA2-R	RT-qPCR	5'- GGAGCTCTTGCACAGGAAAC

RT-qPCR primers were designed using the Primer Express Software installed into the Applied Biosystems PCR System. The sizes of PCR products ranged from 80 to 300 nucleotides in length. F, forward primer; R, reverse primer.

Supplementary Table 2. Primers used in chromatin immunoprecipitation assays.

Primer	Sequence
ROP6(A)-F	ACTCATCATTTTATAACATTTTTCA
ROP6(A)-R	TACAGACAATAAACAATTCAAGGTA
ROP6(B)-F	AAACGTTTTAGAAAAACAGAGATTA
ROP6(B)-R	AAAAAGAAAAAGAAGAGAGTTCAA
ROP6(C)-F	TTTAACGGTCAATAAGTGTTTTTA
ROP6(C)-R	GATTGGAGTGTTAATTTTTATTGA
ROP6(D)-F	GATCTGTGGTCTTCTCCTGA
ROP6(D)-R	AATCAGGCGATTAAGAGTTG
ROP10(E)-F	AAGATTGATGAATAAGTTTGATAGC
ROP10(E)-R	AATAAAACAAATTTTACCTGGTTC
ROP10(F)-F	CGAAATCAATAAATCTTAATCACAT
ROP10(F)-R	TATTTATTTAATAGGGGAATGTACC
ROP10(G)-F	GTTTCAATTTTGTATTTTTGTCTCT
ROP10(G)-R	TTAACAATTGTCTTATAGATTCGTG
ROP10(H)-F	AACCAGCAGTGAAACAAAAG
ROP10(H)-R	GCACAATAGAGAAGCCAATC
ROP11(I)-F	AGCAAATTAAAGAAAACAATAAAAG
ROP11(I)-R	GAAATTATCCATAAAGTGAAAAAGA
ROP11(J)-F	AAATACTAAAATATTGGACCAGAGA
ROP11(J)-R	AGACTCATGGTTTAGAAAGTAAAAG
ROP11(K)-F	TATTTTGTAACCCTAGCTTGTAAGT
ROP11(K)-R	TTACACTTGTATTGGATTGTAAGAA
ROP11(L)-F	GCATTTACCAACAGATTCCT
ROP11(L)-R	CCATCAAGCCTTATAGTGGA

F, forward primer; R, reverse primer.

Supplementary Table 3. Core promoter sequences used for transient expression assays.

KCS1	AATAACTAGAAAAATACTCGAAACTAACTAGA	mKCS1	AAGGGGGGGAAAAATACTCGAAACGGGGGGGA
KCS2	GTCCCTAACTATAACTATAATTC	mKCS2	GTCCCGGGGGGGGGGGGTAATTC
KCS6	AAGATTAACTGTAATTC	mKCS6	AAGATGGGGGGTAATTC
ROP6	TCAATAACTAGCTATCAATAACTAGCTA	mROP6	TCAAGGGGGGGCTATCAAGGGGGGGCTA
ROP10	TCTTTAACTAATTTAAGCTAACTAATAT	mROP10	TCTTGGGGGGATTTAAGCGGGGGGATAT
ROP11	TCTCTAGTTAACAATCTCTAGTTAACAA	mROP11	TCTCGGGGGGACAATCTCGGGGGGACAA

The red sequences indicate core R2R3-MYB binding *cis*-elements.
