# Supplementary material for: MYB96 recruits the HDA15 protein to suppress negative regulators of ABA signaling in Arabidopsis
Source: Nat Commun. 2019 Apr 12;10:1713. doi: 10.1038/s41467-019-09417-1 (PMC6461653; doi:10.1038/s41467-019-09417-1)
Supplement: Supplementary file 2 — Peer Review [file 41467_2019_9417_MOESM2_ESM.pdf]

Reviewers' comments:

Reviewer #1 (Remarks to the Author):

This work by Lee et al. describes that MYB96 associates with HDA15 to repress some of ROP gene expression. Authors identified HDA15 as a MYB96-inetaracting protein by Y2H, BiFC and Co-IP analysis. And then reverse-genetic analysis demonstrated that HDA15 acts as a regulator of ABA response in Arabidopsis. Gene expression or ChIP assays suggested that ROP promoter regions could be targets of MYB96-HDA15 complex. Overall, experiments are well designed, and results are very clear to support their conclusion.

However, there are some problems in this manuscript. The most significant problem is that the relationship between MYB96 and HDA15 is still not confirmed genetically. They used myb96 and HDA15 knockout mutants, or MYB96OX/hda15 transgenic plants, to show their relationship. Why don't you use myb96 hda15 double mutant for phenotypic analysis, gene expression and ChIP analysis? The double mutant will provide concrete evidence for the functional interactions between MYB96 and HDA15 in plants. It will be required for phenotypic analysis, gene expression and ChIP assays.

[Other comments]
1. In Fig. 1c and d, AD-MYB96 and BD-HDA15 were used in Y2H assay. Have you ever swapped AD and BD?

2. In Fig. 1e, Co-IP showed in vivo interaction between MYB96 and HDA15. It is possible that ABA can affect their interactions. Authors should check that. And the resolution of image is too low. High resolution images should be used in this figure.

3. In Fig. 4a, 35S-MYB96/hda15-1 should be required.

4. Methods should be described in detail, for example, how much concentrations, incubation/reaction time, etc. were used for experiments, especially for ChIP assay. Such information must be important to reproduce the results.

[Minor points]
1. P.14, L311-316, descriptions about the MYB96-HDA15 interaction and light signaling is too strong.
2. P.14, L317, comma should be period.4.
3. P.14, L330, What is pBA002?
4. P.16, L382, What is pSATN?
5. P17, What is the vector name used for transient expression analysis?

Reviewer #2 (Remarks to the Author):

Although the manuscript provides interesting data suggesting that MYB96 may recruit the histone deactetylase HDA15 to regulate gene expression involved in ABA signaling in Arabidopsis, the data presented are preliminary and the underlying molecular mechanism is not well defined. Further research is required to define the interaction of HDA15 with MYB96 and their functions in ABA signaling and stress response.

Major points

1. CoIP assays for protein-protein interaction were carried out using N. benthamiana. The authors

need to carry out the CoIP assays in Arabidopsis using the native promoters driven constructs to make sure that the observed protein-protein interaction occurs under nature conditions.

2. More evidence is required to support the authors' claim that MYB96 recruit HDA15 to regulate gene expression. The authors may want to carry out ChIP-seq analysis to compare the global binding of these proteins, which will provide evidence whether they co-target to the overlapping set of genes.

3. The authors also need to generate and analyze the myb96 hda15 double mutant to further investigate the function and interaction of HDA15 and MYB96.

4. ChIP-qPCR analysis showing that MYB96 and HDA15 bind to ROS genes were carried out using 35S:MYB96-MYC and 35S:HDA15-GFP transgenic plants. The authors need to carry out the ChIP-qPCR analysis using the native promoters driven constructs to make sure that MYB96 and HDA15 bind to ROS genes under nature conditions.

5. In Figure 6, the authors can also co-transform p35S::MYB96 and p35S::HDA15 with their reporter constructs in both Col and had15 protoplasts in transient expression assays. This experiment will further confirm whether HDA15 affects MYB96-repressed genes only.

Minor points

1. Figure 1D

The nuclear maker is required to support the claim that MYV96 and HDA15 interacts in the nucleus.

2. Figure 2 E-G

Gene expression was analyzed in seedlings treated with ABA for 6 h, I suggest that the authors may want to analyze more time points such as 12 and 24 h.

3. Figure 5

Since HDA15 affects both histone H3 and H4 deacetylation, the authors may also want to analyze whether H4ac accumulation at the ROP promoters in the myb96 and hda15 mutants was also affected.

4. Page 9 – "Reporter constructs, in which the ROP and KETOACYL-COA SYNTHASE (KCS) promoter sequences were fused with the minimal 35S promoter".

Please explain why the KCS promoter was selected for making reporter constructs.

5. Figure 6

Please explain what are pmKCS and pmROP.

Reviewer #3 (Remarks to the Author):

The work from Lee and Seo describes the interaction between the transcription factor MYB96 and the Histone Deacetylase HDA15 and how this interaction regulates expression of ABA responsive genes as well plant growth under drought conditions.

The premises are interesting, however the manuscript lacks of controls, some results are inconsistent and some conclusions are contradictory.

Figure 1d: a western blot showing similar protein levels in each sample will be informative.

Figure 1e: The IP using an anti-MYC Ab shows a double band which is not present in the input: can the authors please explain that?

Figure 2a: The germination assay shows hyposensitivity to ABA for hda15 mutant and hypersensitivity for HDA15 OX. If these phenotypes depend on MYB96-HDA15 interaction I think it is important to show also results for the myb96 mutant and overexpressing lines.
Figure 2c: Drought experiment shows an opposite phenotype from what the authors show in figure 2a. Can the authors discuss these in more details? Again, the myb96 lines (mutant and OX) are missing in this experiment.

Figure 2g: Although in figure 2f both mutant and OX HDA15 lines are present, for this experiment only mutants are used. I think the data should include the OX HDA15 lines as well as the myb96 mutant and overexpressing lines. From the figure legend I understand that the seedlings were 10 days old. What about testing genes for the germination phenotype? If the opposite effect might be due to the dual function of MYB96 (repressor in some cases and activator in others) testing gene response is important for the model.

Figure 4: Showing ChIP-PCR data of no regulatory regions for each locus will be informative.

Figure 5: The data in this figure are quite inconsistent with each other. In figure A the authors look at the acetylation levels at the ROP transcriptional start sites in the myb96 and hda15 mutants and in their respective overexpressing lines. First there no increase in the acetylation levels for both mutants in control conditions compared to the wild type. This result is already difficult to interpret since in case of loss of histone de-acetylation you would expect an increase in acetylation. Usually histone acetylation positively correlates with gene expression, however in this case there is no correlation as in figure 3 we observe an increase in gene expression in both mutants. Furthermore for the overexpressing lines there is a decrease in acetylation which is consistent with the expression data, however this decrease occurs in different promoter regions, not just on the areas where the authors show binding of MYB96 and HDA15.

To further corroborate the model it would be interesting to see the effect in the double mutant.

Again, if the main point of this manuscript is the role of the interaction between MYB96 and HDA15, each experiment should provide data looking at the effect of both genes.

Responses to Reviewer #1:

The most significant problem is that the relationship between MYB96 and HDA15 is still not confirmed genetically. They used myb96 and HDA15 knockout mutants, or MYB96OX/hda15 transgenic plants, to show their relationship. Why don't you use myb96 hda15 double mutant for phenotypic analysis, gene expression and ChIP analysis? The double mutant will provide concrete evidence for the functional interactions between MYB96 and HDA15 in plants. It will be required for phenotypic analysis, gene expression and ChIP assays.

→ **We generated the *myb96-1hda15-1* double mutant and confirmed the genetic relationship of MYB96 and HDA15, as suggested. We examined seed germination (see Figure 3b), drought tolerance (see Figure 3c), expression of ABA-responsive genes (see Figure 3d), and accumulation of H3/H4 acetylation (see Figure 6a and 6b) in the *myb96-1hda15-1* mutant.**

[Other comments]

1. In Fig. 1c and d, AD-MYB96 and BD-HDA15 were used in Y2H assay. Have you ever swapped AD and BD?

→ **We used the domain-swapped constructs, but it was difficult to see the results, because the BD-MYB96 fusion had strong self-transcriptional activation activity and masked the GAL4 activation induced by physical interactions between MYB96 and HDA15. We thus carried out alternative assays, including BiFC and Co-IP, to confirm the physical interactions. Please understand our reasoning.**

2. In Fig. 1e, Co-IP showed in vivo interaction between MYB96 and HDA15. It is possible that ABA can affect their interactions. Authors should check that. And the resolution of image is too low. High resolution images should be used in this figure.

→ **We repeated the Co-IP assays and replaced the images with high resolution images (see Figure 1e). In addition, we also examined the interaction of MYB96 with HDA15 in the presence of different concentrations of ABA and found that they interact with each other in a dose-dependent manner. The data were newly included as Figure 3a.**

3. In Fig. 4a, 35S-MYB96/hda15-1 should be required.

→ **Binding of MYB96 to the *ROP* loci was examined in *hda15-1* mutant background, as suggested. Please see Figure 5a.**

4. Methods should be described in detail, for example, how much concentrations, incubation/reaction time, etc. were used for experiments, especially for ChIP assay. Such information must be important to reproduce the results.

→ **We described our experiment protocols in more detail in the Materials and Methods section, as suggested.**

[Minor points]
1. P.14, L311-316, descriptions about the MYB96-HDA15 interaction and light signaling is too strong.

→ **We revised and toned-down the sentences to avoid being misleading.**

2. P.14, L317, comma should be period.

→ **We corrected the mistake.**

3. P.14, L330, What is pBA002?

→ **We included a relevant citation to provide information.**

4. P.16, L382, What is pSATN?

→ **We included a relevant citation to provide information.**

5. P17, What is the vector name used for transient expression analysis?

→ **We modified the descriptions and included a citation to provide information.**

Responses to Reviewer #2:

Major points

1. CoIP assays for protein-protein interaction were carried out using N. benthamiana. The authors need to carry out the CoIP assays in Arabidopsis using the native promoters driven constructs to make sure that the observed protein-protein interaction occurs under nature conditions.

→ **The *pMYB96:MYB96-MYC x pHDA15:HDA15-GFP* transgenic *Arabidopsis* plants were generated and used for Co-IP assays. We also examined their physical interactions in the presence of different concentrations of ABA. Please see Figure 3a.**

2. More evidence is required to support the authors' claim that MYB96 recruit HDA15 to regulate gene expression. The authors may want to carry out ChIP-seq analysis to compare the global binding of these proteins, which will provide evidence whether they co-target to the overlapping set of genes.

→ **We fully agree with the reviewer's comment. Global binding analysis of MYB96 and HDA15 and the direct comparison of genome-wide association would add valuable evidence for our conclusion. Genome-wide HDA15 binding in wild-type and *myb96-1* backgrounds may also be relevant. Although we have attempted several times, it was difficult to get a comprehensive view from the global data. Instead, it was increasingly clear that the MYB96-HDA15 interaction is obvious at least at the *ROP* loci. We would like to focus more on this aspect in this manuscript. Please understand our reasoning.**

3. The authors also need to generate and analyze the myb96 hda15 double mutant to further investigate the function and interaction of HDA15 and MYB96.

→ **We generated the *myb96-1hda15-1* double mutant and confirmed the genetic relationship of MYB96 and HDA15, as suggested. We examined seed germination (see Figure 3b), drought tolerance (see Figure 3c), expression of ABA-responsive genes (see Figure 3d), and accumulation of H3/H4 acetylation (see Figure 6a and 6b) in the *myb96-1hda15-1* mutant.**

4. ChIP-qPCR analysis showing that MYB96 and HDA15 bind to ROP genes were carried out using 35S:MYB96-MYC and 35S:HDA15-GFP transgenic plants. The authors need to carry out the ChIP-qPCR analysis using the native promoters driven constructs to make sure that MYB96 and HDA15 bind to ROP genes under nature conditions.

→ **We performed ChIP-qPCR analysis using transgenic plants expressing the native promoter constructs, as suggested. The results were newly included as Supplementary Figure S8.**

5. In Figure 6, the authors can also co-transform p35S::MYB96 and p35S::HDA15 with their reporter constructs in both Col and had15 protoplasts in transient expression assays. This experiment will further confirm whether HDA15 affects MYB96-repressed genes only.

→ **Transient expression assays in the combinations suggested by the reviewer were carried out. We appreciate the suggestion and were able to add additional solid evidence that HDA15 selectively regulates MYB96-repressed genes. The data were newly included in Supplementary Figure S9.**

Minor points

1. Figure 1D, The nuclear maker is required to support the claim that MYV96 and HDA15 interacts in the nucleus.

→ **We replaced the original file with new results containing the nuclear marker. Please see Figure 1d.**

2. Figure 2 E-G, Gene expression was analyzed in seedlings treated with ABA for 6 h, I suggest that the authors may want to analyze more time points such as 12 and 24 h.

→ **We tested additional time points to provide more solid evidence, as suggested. Please see Figure 2g and Supplementary Figure S6.**

3. Figure 5, Since HDA15 affects both histone H3 and H4 deacetylation, the authors may also want to analyze whether H4ac accumulation at the ROP promoters in the myb96 and hda15 mutants was also affected.

→ **We examined both H3 and H4 deacetylation in *myb96-1*, *hda15-1*, and *myb96-1hda15-1* mutants, as suggested. Please see Figure 6a and 6b.**

4. Page 9 – "Reporter constructs, in which the ROP and KETOACYL-COA SYNTHASE (KCS) promoter sequences were fused with the minimal 35S promoter". Please explain why the KCS promoter was selected for making reporter constructs.

→ **The *KCS* genes are known to be directly activated by MYB96. Thus, we suspected that comparison of the *KCS* and *ROP* genes is a good model to show how MYB96 facilitates opposite roles in gene regulation. A concise explanation was provided to address this issue. Please see page 11, 2nd paragraph.**

5. Figure 6, Please explain what are pmKCS and pmROP.

→ **We provided relevant descriptions in the Figure legends section for proper understanding.**

<u>Responses to Reviewer #3:</u>

Figure 1d: a western blot showing similar protein levels in each sample will be informative.

→ **We set up a reliable protocol and are convinced of our transfection efficiency and analysis. We observed the results in the most of the transfected protoplast cells. Please understand our situation.**

Figure 1e: The IP using an anti-MYC Ab shows a double band which is not present in the input: can the authors please explain that?

→ **We performed the Co-IP assays several times and optimized the experimental conditions. The results were replaced to avoid being misleading. Please see Figure 1e.**

Figure 2a: The germination assay shows hyposensitivity to ABA for hda15 mutant and hypersensitivity for HDA15 OX. If these phenotypes depend on MYB96-HDA15 interaction I think it is important to show also results for the myb96 mutant and overexpressing lines.

→ **The germination assays for *MYB96-ox* and *myb96-1* mutants were included, as suggested. Please see Figure 2a and 2b.**

Figure 2c: Drought experiment shows an opposite phenotype from what the authors show in figure 2a. Can the authors discuss these in more details? Again, the myb96 lines (mutant and OX) are missing in this experiment.

→ **The drought tolerance assays for *MYB96-ox* and *myb96-1* mutants were included, as suggested (Please see Supplementary Figure S5). Please note that ABA hypersensitive responses include delayed seed germination and increased drought tolerance, whereas ABA hyposensitive phenotypes are accelerated seed germination and drought susceptibility. Therefore, Figure 2a and 2c are NOT opposite phenotypes. The seed germination and drought responses are clearly attributable to altered ABA sensitivity.**

Figure 2g: Although in figure 2f both mutant and OX HDA15 lines are present, for this experiment only mutants are used. I think the data should include the OX HDA15 lines as well as the myb96 mutant and overexpressing lines. From the figure legend I understand that the seedlings were 10 days old. What about testing genes for the germination phenotype? If the opposite effect might be due to the dual function of MYB96 (repressor in some cases and activator in others) testing gene response is important for the model.

→ **We additionally included *HDA15-ox*, *myb96-1*, and *MYB96-ox* plants to validate our conclusions (please see Figure 2g). Germination and drought phenotypes of *HDA15*- and *MYB96*-misexpressing transgenic plants are all attributable to altered sensitivity to ABA, and expression of ABA-responsive genes is an important clue for our conclusion. Please understand our reasoning.**

Figure 4: Showing ChIP-PCR data of no regulatory regions for each locus will be informative.

→ **The MYB96-HDA15 complex primarily binds to the promoter regions. We thus included a gene body region as a negative control, as suggested. We also tested H3ac and H4ac levels at the control region for each locus. Please see Figure 5a, 5b, 6a, and 6b, and Supplementary Figure S8.**

Figure 5: The data in this figure are quite inconsistent with each other. In figure A the authors look at the acetylation levels at the ROP transcriptional start sites in the myb96 and hda15 mutants and in their respective overexpressing lines. First there no increase in the acetylation levels for both mutants in control conditions compared to the wild type. This result is already

difficult to interpret since in case of loss of histone de-acetylation you would expect an increase in acetylation. Usually histone acetylation positively correlates with gene expression, however in this case there is no correlation as in figure 3 we observe an increase in gene expression in both mutants. Furthermore for the overexpressing lines there is a decrease in acetylation which is consistent with the expression data, however this decrease occurs in different promoter regions, not just on the areas where the authors show binding of MYB96 and HDA15. To further corroborate the model it would be interesting to see the effect in the double mutant.

→ **Thank you very much for your suggestion. To overcome the inconsistency, we performed more experiments with 8 biological replicates. The original results were replaced with the final data. Since H4 acetylation levels are also related to gene expression and H4 acetylation is also influenced by HDA15, we also included data showing accumulation of H4 acetylation at each locus. We also analyzed the *myb96-1hda15-1* double mutant, as suggested. In addition, we intensively compared the MYB96/HDA15-binding regions and control regions of the *ROP* loci to build a solid conclusion. Please see Figures 6a, 6b, 6c, and 7c.**

Reviewers' comments:

Reviewer #1 (Remarks to the Author):

Authors significantly revised the manuscript and well answered to all points raised by this reviewer.

Reviewer #2 (Remarks to the Author):

The revised manuscript has addressed most of my previous concerns. I have the following suggestions.

1. Figure 1b – Please indicate what the red bars and blue bars represent in the gene construct diagram. The authors claimed that an N-terminal fragment of MYB96 containing the R2R3-MYB DNA-binding domainis responsible for interaction with the N-terminal fragment of HDA15. Dose the N-terminal fragment of HDA15contin any specific domain that may be important for this interaction?

2. There seems to be additive effects between myb96 and hda15 on seed germination and plant survival phenotypes (Fig. 3b and 3c). However, the additive effect was not observed in the expression and acetylation of ROPs (Fig 4, 6). This needs to be explained and discussed.

3. In Figure 6c, it is understandable that the H3ac level and expression of ROP genes are reduced in 35S:HDA15-GFP plants. However, why are the H3ac level and expression of ROP genes increased in 35S:HDA15-GFP/myb96-1 plants compared to wild type? Please explain.

4. Figure 7c is redundant with Figure 6c and can be removed.

5. How pmKCS and pmROP mutant constructs were generated need to be described in the method section.

6. Page 11, line 261 – "Taken together, the MYB96-HDA15 complex represses the ROPs, which are negative regulators of ABA signaling, to pro 262 mote ABA responses. The ABA-inducible MYB96 protein binds to the promoters of ROP genes and recruits HDA15 to facilitate H3 and H4 deacetylation. In the presence of high concentrations of ABA, the ROP genes are repressed, concomitant with histone deacetylation, in a MYB96- and HDA15-dependent pathway to ensure full activation of ABA responses (Fig. 8)".

This part should be moved to the discussion section.

Reviewer #3 (Remarks to the Author):

The manuscript is much improved compared to the previous version and the authors addressed some of my previous concerns. However there is big discrepancy between the data submitted in the former and current version that the authors have not yet addressed.

Figure 1: If you can't provide a western blot for the transfected cells, it will be informative to have a western blot for the yeast interaction.

Figure 1E: I appreciate that the western has been repeated but an explanation for the previous results is still required, particularly for the presence of a double band.

Figure 2: if the effects of ABA during germination and drought tolerance are both due to altered ABA sensitivity, it is possible that HDA15 and MYB96 are involved in different ABA response pathways. Therefore it is important to investigate the molecular mechanism in both pathways. It is not clear to me at what developmental stage the expression of RD22, RD29A and COR47 was assessed and how it relates with the different phenotypic responses.

Figure 6: I still have major issues with this figure. First of all the authors replaced the original results with the final data without addressing the discrepancies with the previous version. Furthermore the data from the double mutant phenotype clearly showed an additive effect that is not visible in terms of gene expression in figure 4 and acetylation. Compared to the previous version where the authors investigated the acetylation levels of the regions A-B-C for each locus, now they show data for C-D region, ignoring the changes in acetylation that were previously observed in region B.

<u>Responses to Reviewer 2</u>

1. Figure 1b – Please indicate what the red bars and blue bars represent in the gene construct diagram. The authors claimed that an N-terminal fragment of MYB96 containing the R2R3-MYB DNA-binding domainis responsible for interaction with the N-terminal fragment of HDA15. Dose the N-terminal fragment of HDA15 contin any specific domain that may be important for this interaction?

→ **We provided more detailed information obtained from multiple public databases. Please see Figure 1b, figure legends, and revised manuscript (page 5, 2nd paragraph).**

2. There seems to be additive effects between myb96 and hda15 on seed germination and plant survival phenotypes (Fig. 3b and 3c). However, the additive effect was not observed in the expression and acetylation of ROPs (Fig 4, 6). This needs to be explained and discussed.

→ **MYB96 alone can regulate ABA-inducible genes (e.g. KCSs), and likewise HDA15 may also have additional functions in ABA responses indepedently of MYB96. This may explain additive effects of *myb96-1hda15-1* on general ABA responses. However, at least in the regulation of *ROP* genes, both MYB96 and HDA15 are required and consistenly, no additive effects on the gene regulation were observed. We described the situation in the revised manuscript: please see page 9, 1st paragraph.**

3. In Figure 6c, it is understandable that the H3ac level and expression of ROP genes are reduced in 35S:HDA15-GFP plants. However, why are the H3ac level and expression of ROP genes increased in 35S:HDA15-GFP/myb96-1 plants compared to wild type? Please explain.

→ **HDA15 regulation of acetylation and expression of *ROP*s depends on MYB96, because MYB96 specifies promoter regions of *ROP* loci. HDA15 would be less recruited in *myb96-1* compared to wild type (low level of MYB96 will allow recruitment of HDA15 in WT) even under normal growth condition, and thus H3ac levels and transcript accumulation were further increased, similar to *myb96-1* mutant (please see Figure 4c, 6a, and 6b).**

4. Figure 7c is redundant with Figure 6c and can be removed.

→ **Figure 6c shows dependence of HDA15 on MYB96 in DNA binding, whereas Figure 7c shows dependence of MYB96 on HDA15 in histone deacetylation-based gene repression. While they look similar, we carefully think that they have different meaning. Therefore, we would like to maintain current organization. Please understand our reasoning.**

5. How pmKCS and pmROP mutant constructs were generated need to be described in the method section.

→ **We provided details about pmKCS and pmROP constructs in the Method section and Supplemetentary Table S3.**

6. Page 11, line 261 – “Taken together, the MYB96-HDA15 complex represses the ROPs, which are negative regulators of ABA signaling, to pro 262 mote ABA responses. The ABA-inducible MYB96 protein binds to the promoters of ROP genes and recruits HDA15 to facilitate H3 and H4 deacetylation. In the presence of high concentrations of

ABA, the ROP genes are repressed, concomitant with histone deacetylation, in a MYB96- and HDA15-dependent pathway to ensure full activation of ABA responses (Fig. 8)". This part should be moved to the discussion section.

→ **We reorganized the descriptions in the Discussion section.**

<u>Responses to Reviewer 3</u>

1. Figure 1: If you can't provide a western blot for the transfected cells, it will be informative to have a western blot for the yeast interaction.

→ **Protoplasts transfected with the BiFC constructs were harvested to perform western blot analysis. Full-sized proteins were well-expressed, and similar protein levels were detected in each transfected sample. Please see Supplementary Figure S3.**

2. Figure 1E: I appreciate that the western has been repeated but an explanation for the previous results is still required, particularly for the presence of a double band.

→ **Posttranslational modifications of the proteins might be involved, based on the fact that multiple bands are usually detected from epitope-tagged MYB96 and HDA15. Physical interactions most likely do not depend on certain types of protein modifications. Please understand our reasoning.**

3. Figure 2: if the effects of ABA during germination and drought tolerance are both due to altered ABA sensitivity, it is possible that HDA15 and MYB96 are involved in different ABA response pathways. Therefore it is important to investigate the molecular mechanism in both pathways. It is not clear to me at what developmental stage the expression of RD22, RD29A and COR47 was assessed and how it relates with the different phenotypic responses.

→ **In general, if ABA signaling is activated at an upstream point of the pathways, delayed seed germination and enhanced drought tolerance both can be simultaneously induced, as exemplified by overexpression of ABA receptors and SnRKs or mutations in PP2C genes (abi). Considering that ROPs are upstream components of ABA signaling, which act with PP2Cs, the MYB96-HDA15-ROP module most likely regulates diverse ABA responses by promoting the upstream ABA signaling. Also, we included transcript accumulation of ABA signaling marker genes *RD22, RD29A*, and *COR47* at the stage of early seedlings to support our claim that the MYB96-HDA15-ROP module is critical for enhancing ABA signaling, regardless of plant developmental stages (Please see Supplementary Figure S8). As the reviewer indicated, we cannot rule out that the MYB96 and HDA15 proteins may have additional, specific roles in ABA-dependent seed germination and stomatal movement for drought tolerance, but now we would like to emphasize this study that focuses on general ABA responses modulated by the MYB96-HDA15 complex acting at an upstream point.**

4. Figure 6: I still have major issues with this figure. First of all the authors replaced the original results with the final data without addressing the discrepancies with the previous version. Furthermore the data from the double mutant phenotype clearly showed an additive effect that is not visible in terms of gene expression in figure 4 and acetylation. Compared to the previous version where the authors investigated the acetylation levels of the regions A-B-C for each locus, now they show data for C-D region, ignoring the changes in acetylation that were previously observed in region B.

→ Regarding Figure 6, in the previous submission, we chose three promoter regions to check histone acetylation levels. As shown before, they all have similar trends, which may be due to expansion and propagation of chromatin contexts. To improve the quality of the data, we needed to provide negative controls (3'end of gene) to show that ABA-induced changes in H3ac/H4ac levels at *ROP* loci are particularly observed at the promoters. In addition, we also followed higher standard of ChIP assays by providing absolute values, rather than relative enrichment. By replacing the data, we could tell the regional specificity of histone modification with increased accuracy. This is why we updated the figures. To address the reviewer's comment, we included ChIP data conducted on another promoter region (which was included in previous submission, but updated with more replicates in the current manuscript). Please see Supplementary Figure S11.

In addition, we would like to make interpretation about double mutant phenotypes. In addition to ROPs, MYB96 alone can regulate ABA-inducible genes (Figure 7 and other papers), and likewise HDA15 may also have additional functions in ABA responses indepedently of MYB96. This may explain additive effects of *myb96-1hda15-1* on general ABA responses. However, at least in the 'regulation of *ROP* genes', both MYB96 and HDA15 are required and consistenly, no additive effect on the gene regulation was observed. It is reasonble that ROP genes are not sole regulatory targets of MYB96 and HDA15 for the control of ABA responses. We described the situation in the revised manuscript: please see page 9, 1st paragraph.

Reviewers' comments:

Reviewer #2 (Remarks to the Author):

My previous concerns have been addressed in the revised manuscript.

Reviewer #3 (Remarks to the Author):

This manuscript focuses on the physiological significance of the interaction between MYB96 and HDA15 in regulating ABA responses,therefore more emphasis should be addressed on the outputs derived from this interaction. Furthermore it would be beneficial to discuss the putative responses that are not regulated by such interaction but primarily by each individual member. In such context, while the role of MYB96 in mild ABA responses is described in the model, there is no mention of HDA15 in that response.

The data presented suggests that the interaction between MYB96 and HDA15 is also occurring under control conditions, yet the model indicates that such interaction is only required under high ABA conditions.
It will be interesting to monitor whether HDA15 expression is also induced by abiotic stress.

Pil Joon Seo, Ph.D.
Department of Chemistry
Seoul National University
Seoul 08826
Korea

Responses to Reviewer 3

1. This manuscript focuses on the physiological significance of the interaction between MYB96 and HDA15 in regulating ABA responses, therefore more emphasis should be addressed on the outputs derived from this interaction. Furthermore it would be beneficial to discuss the putative responses that are not regulated by such interaction but primarily by each individual member. In such context, while the role of MYB96 in mild ABA responses is described in the model, there is no mention of HDA15 in that response.

→ **We tried to further emphasize the relevance of MYB96-HDA15 interactions in ABA responses. Please see page 14, last paragraph; page 15, 1st paragraph. Thank you for the suggestion.**

2. The data presented suggests that the interaction between MYB96 and HDA15 is also occurring under control conditions, yet the model indicates that such interaction is only required under high ABA conditions. It will be interesting to monitor whether HDA15 expression is also induced by abiotic stress.

→ **We examined effects of several abiotic stress factors on *HDA15* expression, and the data were included as Supplementary Figure S7. We also complemented our descriptions in the revision.**

REVIEWERS' COMMENTS:

Reviewer #3 (Remarks to the Author):

In the current revised version, the authors addressed the previous comments.
